# Double exponential quadrature for fractional diffusion

**DOI:** 10.1007/s00211-022-01342-8

**Published:** 2023-01-26

**Authors:** Alexander Rieder

**Affiliations:** 1 Fakultät für Mathematik, University of Vienna, Vienna, Austria; 2 Institute for Analysis and Scientific Computing, TU Wien, Vienna, Austria

**Keywords:** 65N15, 65M12

## Abstract

We introduce a novel discretization technique for both elliptic and parabolic fractional diffusion problems based on double exponential quadrature formulas and the Riesz–Dunford functional calculus. Compared to related schemes, the new method provides faster convergence with fewer parameters that need to be adjusted to the problem. The scheme takes advantage of any additional smoothness in the problem without requiring a-priori knowledge to tune parameters appropriately. We prove rigorous convergence results for both, the case of finite regularity data as well as for data in certain Gevrey-type classes. We confirm our findings with numerical tests.

## Introduction

The study of processes governed by fractional linear operators has gathered significant interest over the last few years [8, 22, 35] with applications ranging from physics [1] to image processing [1, 15, 16], inverse problems [19] and more. See [33] for an overview of applications in different fields. The goal is to solve problems of the form

$$\begin{aligned} (-\Delta )^{\beta } u=f \qquad \text {or} \qquad \partial _t^{\alpha } u + (-\Delta )^{\beta }u=f \end{aligned}$$

with parameters $\beta$,$\alpha \in (0,1]$. There are multiple (non-equivalent) ways of defining fractional powers of operators. We mention the integral fractional Laplacian and the spectral definition [22]. In this paper, we focus on the spectral definition which is equivalent to the functional calculus definition.

For discretization of such problems, both stationary and time dependent, multiple approaches have been presented. A summary of the most common can be found in [2, 22]. They can be broadly distinguished into three categories. The first class of methods uses the Caffarelli-Silvestre extension to reformulate the problem as a PDE posed in one additional spatial dimension. This problem is then treated by standard finite element techniques [6, 24, 25, 27–29]. The second big class of discretization schemes, and the one our new scheme is part of, was first introduced in [7] and later extended to more general operators [5] and time dependent problems [3, 4, 26]. They are based on the Riesz–Dunford calculus (sometimes also referred to as Dunford-Taylor or Riesz-Taylor) and employ a $\textrm{sinc}$ quadrature scheme to discretize the appearing contour integral. $\textrm{sinc}$ quadrature, and overall $\textrm{sinc}$-based numerical methods are less well known than their polynomial based counterparts, but provide rapidly converging schemes [21, 32] with very easy implementation. The quadrature relies on appropriate coordinate transforms in order to yield analytic, rapidly decaying integrands over the real line and then discretization using the trapezoidal quadrature rule. In [34] it was realized that by adding an additional $\sinh$-transformation, it is possible to get an even faster convergence for certain integrals. Namely, writing $\mathcal {N}_{\text {q}}$ for the number of quadrature points, instead of convergence of the form $e^{-\sqrt{\mathcal {N}_{\text {q}}}}$, it is possible to get rid of the square root and obtain rates of the form $e^{-\frac{\mathcal {N}_{\text {q}}}{\ln {\mathcal {N}_{\text {q}}}}}$. Further developments in this direction are summarized in [23]. Such schemes are commonly referred to as double exponential quadrature or $\sinh$-${\text {tanh}}$ quadrature. Thirdly there is the large class of methods based on rational approximation of the functions $z^{-\beta }$ and the Mittag Leffler-Function $e_{\gamma ,\mu }(z)$ (see (3.18) for the precise definition). As shown in [17], this class also encompasses the previous two approaches while also allowing some other methods, based on general rational approximation algorithms like Best-Uniform-Rational approximation (BURA) or the “Adaptive Antoulas-Anderson”-algorithm (AAA) from [30]. Finally, there exist some further methods based on reduced basis and rational Krylov methods [9, 10, 12, 13] which are strongly related to rational approximation.

In this paper we investigate whether the discretization of the Riesz–Dunford integral can benefit from using a double exponential quadrature scheme instead of the more established $\textrm{sinc}$-quadrature. We present a scheme that retains all the advantages of [3–5] while delivering improved convergence rates. Namely, the scheme is very easy to implement if a solver for elliptic finite element problems is available. It is almost trivially parallelizable, as the main cost consists of solving a series of independent elliptic problems. In addition, it provides (compared to $\textrm{sinc}$-methods) superior accuracy over a wide range of applications and does not require subtle tweaking of parameters in order to get good performance. Instead it will automatically pick up any additional smoothness of the underlying problem to give improved convergence. Since for each quadrature point an elliptic FEM problem needs to be solved, reducing the number of quadrature points greatly increases performance of the overall method.

Compared to the BURA and AAA rational approximation methods, the sinc- and double-exponential quadrature based algorithms have several advantages. Firstly, the implementation is very simple with quadrature nodes that are known explicitly. The quadrature points are also independent of the spectrum of the operator $\mathcal {L}$ and no explicit bound on the largest eigenvalue is required. This makes them better suited for highly accurate but highly ill-conditioned discretizations like the *hp*-FEM scheme in [26].

Secondly, the quadrature points are independent of the function that is to be approximated. Most notably, when considering the time-dependent problem with inhomogeneus right-hand side in Sect. 3.3, all the linear systems that need to be solved are independent of the time *t* or the integration variable $\tau$. This makes the full time-dependent problem of the same cost (with respect to the number of systems that need solving) as the simple stationary problem. Thirdly, the quadrature based methods allow for very detailed analysis, as showcased in this article. In addition to the quadrature analysis, they also allow for detailed analysis of the error brought in by a discretization in space [26]. In practice, we also observed better numerical stability in the presence of rounding errors, as showcased in Fig. 4.

The paper is structured as follows. After fixing the model problem and notation in Sects. 1.1, 3 introduces the double exponential formulas in an abstract way and we collect some known properties. In addition, we provide one small convergence result which, to our knowledge, has not yet appeared in the literature; we show that the double exponential formulas at least provide comparable convergence of order $e^{-\sqrt{\mathcal {N}_{\text {q}}}}$ even without requiring additional analyticity compared to standard $\textrm{sinc}$ methods.

The paper is structured as follows. In Sect. 1, we introduce the general setting and the functional calculus. Sect. 2 introduces the quadrature scheme as well as the model problems we are interested in. We also state the main convergence results. Sect. 3 is devoted to proving these results. Sect. 3.1 presents the abstract analysis for sinc methods and collects some known properties. In addition, we provide one small convergence result which, to our knowledge, has not yet appeared in the literature; we show that the double exponential formulas at least provide comparable convergence of order $e^{-\sqrt{\mathcal {N}_{\text {q}}/ \ln (\mathcal {N}_{\text {q}})}}$ even without requiring additional analyticity compared to standard sinc methods. In Sect. 3.2, we look at the case of a purely elliptic problem without time dependence. It will showcase the techniques used and provide the building block for the more involved problems later on. In Sect. 3.3, we then consider what happens if we move into the time-dependent regime. Section 4 provides extensive numerical evidence supporting the theory. We also compare our new method to the standard $\textrm{sinc}$-based methods. Finally, Appendix A collects some properties of the coordinate transform involved. The proofs and calculations are elementary but somewhat lengthy and thus have been relegated to the appendix in order to not impact readability of the article.

Throughout this work we will encounter two types of error terms. For those of the form $e^{-\frac{\gamma }{k}}$ we will be content with not working out the constants $\gamma$ explicitly. For the more important terms of the form $e^{-\frac{\gamma '}{\sqrt{k}}}$ we will derive explicit constants $\gamma '$ which prove sharp in several examples of Sect. 4.

We close with a remark on notation. Throughout this text, we write $A \lesssim B$ to mean that there exists a constant $C>0$, which is independent of the main quantities of interest like number of quadrature points $\mathcal {N}_{\text {q}}$ or step size *k* such that $A \le C B$. The detailed dependencies of *C* are specified in the context. We write $A \sim B$ to mean $A \lesssim B$ and $B \lesssim A$.

### General setting and notation

In this paper, we consider problems of applying holomorphic functions *f* to self-adjoint operators, for example the Laplacian. The two large classes of problems treated in this paper stem from the study of fractional diffusion problems, both in the stationary as well as in the transient version. Since it does not incur additional difficulty compared to the explicit setting of Remark 1.2, we will work in the following abstract setting:

#### Assumption 1.1

Let ${\mathcal {X}}$ be a Hilbert space and $\mathcal {L}$ be a positive definite, self adjoint operator on ${\mathcal {X}}$ such that there exists a sequence of eigenvalues $\lambda _j >0$ with associated eigenfunctions $\varphi _j \in {\mathcal {X}}$, $j\in \mathbb {N}_0$, such that $(\varphi _j)_{j=0}^{\infty }$ is an orthonormal basis of ${\mathcal {X}}$.

Given the eigenvalues and eigenfunctions of $\mathcal {L}$, we define the spaces for $\beta \ge 0$

1.1 $$\begin{aligned} {\mathbb {H}}^{\beta }:=\Big \{ u \in {\mathcal {X}}: \left\| u\right\| _{{\mathbb {H}}^\beta (\Omega )} <\infty \Big \} \quad \text {with} \quad \left\| u\right\| ^2_{{\mathbb {H}}^{\beta }}:=\sum _{j=0}^{\infty }{\lambda _j^{\beta } \left| (u,v_j)_{{\mathcal {X}}}\right| ^2}. \end{aligned}$$

#### Remark 1.2

The problem we have in mind for our applications is the following: given a bounded Lipschitz domain $\Omega$, we consider the space ${\mathcal {X}}:=L^2(\Omega )$ and the self adjoint operator

$$\begin{aligned} \mathcal {L}u:=-{\text {div}}(\mathfrak {A} \nabla u) + \mathfrak {c} u, \end{aligned}$$

where $\mathfrak {A} \in L^{\infty }(\Omega ;\mathbb {R}^{d\times d})$ is uniformly symmetric and positive definite and $\mathfrak {c} \in L^{\infty }(\Omega )$ satisfies $\mathfrak {c}\ge 0$ almost everywhere. The domain of ${\text {dom}}(\mathcal {L})$ is always taken to include homogeneous Dirichlet boundary conditions. In this case, the spaces ${\mathbb {H}}^{\beta }({\mathcal {X}})$ correspond to the standard (fractional) Sobolev spaces often denoted by ${\mathbb {H}}^{\beta }(\Omega )$ or $\widetilde{H}^{\beta }(\Omega )$ in the literature. $\square$

#### Remark 1.3

[5] considers an even more general class of operators, namely the class of “regular accretive operators”. We expect some of the results of this article to carry over also to such a class, but since many of our proofs rely on the decomposition using real eigenvalues, such generalizations would be non-trivial. $\square$

The spaces ${\mathbb {H}}^{\beta }$ are the natural setting for our regularity assumptions on the data. If we are interested in convergence beyond root-exponential rates, we need the following class of functions of Gevrey-type.

1.2 $$\begin{aligned} \mathcal {G}^{\mathcal {L}}(C_f,R_f,\omega ):=\Big \{ f \in {\mathcal {X}}: \quad \left\| f\right\| _{{\mathbb {H}}^{\rho }}&\le C_{f} \, R_f^\rho \,\big (\Gamma (\rho +1)\big )^{\omega } < \infty \quad \forall \rho \ge 0 \Big \}. \end{aligned}$$

Compared to the standard Gevrey-class of functions, these spaces also include boundary conditions for the functions $\mathcal {L}^{n} f$ for all $n \in \mathbb {N}$. If the boundary conditions are met, we can then estimate

$$\begin{aligned} \left\| f\right\| _{{\mathbb {H}}^{\rho }}\le \Vert f\Vert _{{\mathcal {X}}}+ \Vert \mathcal {L}^{\lceil \rho /2\rceil } f\Vert _{{\mathcal {X}}}. \end{aligned}$$

Examples for such functions are those only containing a finite number of frequencies when decomposed into the eigenbasis of $\mathcal {L}$, but also more complex functions such as smooth bump functions with compact support are admissible (see [31, Section 1.4]).

One natural way of defining a functional calculus for the operator $\mathcal {L}$ is based on the spectral decomposition.

#### Definition 1.4

(*Spectral calculus*) Let ${\mathcal {O}} \subseteq \mathbb {R}_+$ such that ${\mathcal {O}}$ contains the spectrum of $\mathcal {L}$. Let $g: {\mathcal {O}} \rightarrow \mathbb {C}$ be continuous with $\left| g(z)\right| \lesssim (1+\left| z\right| )^{\mu }$ for $\mu \in \mathbb {R}$. We define for $u \in {\mathbb {H}}^{2\mu }(\Omega )$:

$$\begin{aligned} g(\mathcal {L})u:=\sum _{j=0}^{\infty }{g(\lambda _j) \big (u,v_j\big )_{{\mathcal {X}}} \, v_j }. \end{aligned}$$

An alternative definition for holomorphic functions, which will prove more useful for approximation is given in the following Definition. For simplicity, we restrict our considerations to decaying functions *g*. In this case, it can be shown (see also [3, Section 2]) that the operators resulting from Definitions 1.4 and 1.5 coincide.

#### Definition 1.5

(*Riesz–Dunford calculus*) Fix parameters $\sigma =1/2$ or $\sigma =1$, $\theta \ge 1$ and $\kappa >0$. Let ${\mathcal {O}} \subseteq \mathbb {C}$ such that $\mathbb {C}_{+}:=\{ z \in \mathbb {C}: {\text {Re}}(z)>0\} \subseteq {\mathcal {O}}$. Let $g: {\mathcal {O}} \rightarrow \mathbb {C}$ be holomorphic with $\left| g(z)\right| \lesssim (1+\left| z\right| )^{\mu }$ for $\mu < 0$. We define

1.3 $$\begin{aligned} g(\mathcal {L}):=\frac{1}{2\pi i}\int _{\mathcal {C}}{g(z)\big (\mathcal {L}-z\big )^{-1} \,dz}, \end{aligned}$$

where the integral is taken in the sense of Riemann, and $\mathcal {C}$ is the smooth path

$$\begin{aligned} \mathcal {C}:=\Big \{\kappa \big (\cosh (\sigma w) + i\theta \sinh (w)\big ) \quad \text { for } w \in \mathbb {R}\Big \}. \end{aligned}$$

The parameter $\kappa >0$ is taken sufficiently small such that $\kappa <\lambda _0$, where $\lambda _0$ is the smallest eigenvalue of $\mathcal {L}$. The parameters $\sigma$ and $\theta$ can be used to tweak the discretization. We have observed the best behavior for $\sigma :=1/2$ and $\theta :=4$; cf. Sect. 4.

#### Remark 1.6

The choice of path in Definition 1.5 is somewhat arbitrary. It is only required to encircle the spectrum of $\mathcal {L}$ with winding number 1. Throughout this paper, we will only ever use the same path and thus make it part of our definition. $\square$

#### Remark 1.7

One could also think to allow $\sigma \in (0,1)$. For the practical application of the scheme this does not make a big difference, but the analysis for $\sigma \ne 1$ in this paper makes heavy use of the half-angle formula. Therefore we restrict our view to the cases $\sigma =1$ or $\sigma =1/2$. In numerical experiments, methods with $\sigma \ne 1/2$ work, but we decided that the small difference in performance does not warrant the much greater complexity of analysis. $\square$

## Model problems, discretization and results

In this section, we introduce the discretization methods and, in order to ease the reading of the article, we present the most important of the convergence results. All of the sometimes very technical proofs are relegated to Sect. 3.

The main role in our discretization schemes will be played by the following coordinate transform which parametrizes the contour in Definition 1.5:

2.1 $$\begin{aligned} \psi _{\theta ,\sigma }(y):=\kappa \Big [\cosh \Big (\frac{\sigma \pi }{2} \sinh (y)\Big ) + i\theta \sinh \Big (\frac{\pi }{2} \sinh (y)\Big )\Big ]. \end{aligned}$$

We will focus on the cases $\sigma \in \big \{\frac{1}{2},1 \big \}$ and $\theta \ge 1$. $\kappa$ is again taken sufficiently small as in Definition 1.5.

Using this transformation, we can introduce the double exponential quadrature approximation of the Riesz–Dunford calculus in Definition 1.5. Because the discretization by quadrature will appear repeatedly for different functions and operators, we introduce the following notation:

### Definition 2.1

Let ${\mathcal {O}}\subseteq \mathbb {C}$ such that $\mathbb {C}_+ \subseteq {\mathcal {O}}$. For $g: {\mathcal {O}} \rightarrow \mathbb {C}$ holomorphic as in Definition 1.5, $k \ge 0$ and $\mathcal {N}_{\text {q}}\in \mathbb {N}\cup \{\infty \}$ we write for all $u \in {\mathcal {X}}$

2.2 $$\begin{aligned} Q^{\mathcal {L}}(g,\mathcal {N}_{\text {q}})u&:=\frac{1}{2\pi i} \sum _{j=-\mathcal {N}_{\text {q}}}^{\mathcal {N}_{\text {q}}}{g(\psi _{\sigma ,\theta }(j k)) \psi _{\sigma ,\theta }'( j k)(\mathcal {L}- \psi _{\sigma ,\theta }(j k))^{-1} u} \end{aligned}$$

and $Q^{\mathcal {L}}(g):=Q^{\mathcal {L}}(g,\infty )$ for the case where no cutoff is performed. The quadrature error will be denoted by

2.3 $$\begin{aligned} E^{\mathcal {L}}(g,\mathcal {N}_{\text {q}})&:=g(\mathcal {L})u - Q^{\mathcal {L}}(g,\mathcal {N}_{\text {q}})u, \qquad \forall u \in {\text {dom}}(g(\mathcal {L})) \end{aligned}$$

where $g(\mathcal {L})$ is given via the Riesz–Dunford integral 1.5. Again, we write $E^{\mathcal {L}}(g):=E^{\mathcal {L}}(g,\infty )$.

### Remark 2.2

In Definition 2.1, we will often work with the special case $\mathcal {L}=\lambda$. This is taken to mean the scalar multiplication operator $u \mapsto \lambda u$ on the vector space ${\mathcal {X}}$. $\square$

We apply the function to the following problems:

(i) $g(z):=z^{-\beta }$: This corresponds to solving an elliptic fractional diffusion problem; see Sect. 2.1 for the model problem and Sect. 3.2 for the proofs.
(ii) $g(z)=e_{\gamma ,\mu }(-t^\alpha z^{\beta })$, with $e_{\gamma ,\mu }$ the Mittag–Leffler function: This corresponds to a parabolic model problem; see Sects. 2.2 and 3.3.

For both model problems, we prove two convergence results, depending on the regularity of the data. In the case of “finite regularity”, the data (*f* or $u_0$) are assumed to be in a space ${\mathbb {H}}^{2\rho }$ for some $\rho >0$. This results in bounds of root-exponential order $\sqrt{\mathcal {N}_{\text {q}}/\ln (\mathcal {N}_{\text {q}})}$.

The second case is the one were the data are in the Gevrey-type classes $\mathcal {G}^{\mathcal {L}}$ introduced in (1.2). For such functions, the double-exponential discretization leads to an improved convergence of the form $\mathcal {O}(e^{-\frac{\gamma \mathcal {N}_q}{\ln (\mathcal {N}_q)}})$.

### The elliptic problem

As our first model problem, we consider the following elliptic fractional diffusion problem:

2.4 $$\begin{aligned} \text {Given}\, f \in L^2(\Omega )\, \text {and}\, \beta >0, \; \text {find}\, u \in {\text {dom}}(\mathcal {L}^\beta )\, \text {such that } \;\; \mathcal {L}^{\beta } u=f. \end{aligned}$$

Using the Riesz–Dunford formula, this is equivalent to computing

$$\begin{aligned} u&=\frac{1}{2\pi i} \int _{\mathcal {C}}{z^{-\beta }\big (\mathcal {L}-z\big )^{-1} f\,dz}. \end{aligned}$$

In order to get a discrete scheme, we replace the integral with the quadrature formula. Given $\mathcal {N}_{\text {q}}\in \mathbb {N}$ and $k >0$, the approximation to (2.4) is then given by

$$\begin{aligned} u_k:=Q^\mathcal {L}(z^{-\beta },\mathcal {N}_{\text {q}}) f. \end{aligned}$$

#### Remark 2.3

Since in practice, the solution operator $(\mathcal {L}-z)^{-1}$ is not computable, one would in addition replace $(\mathcal {L}-z)^{-1}$ by a Galerkin solver in order to obtain a fully computable scheme. In the Setting of Remark 1.2, this means the following: given a closed subspace $\mathbb {V}_{h}\subseteq H_0^1(\Omega )$, the discrete resolvent $R_h(z): L^2(\Omega )\rightarrow \mathbb {V}_{h}$ is given as the solution

$$\begin{aligned} R_h(z) f:=u_h, \; \text {with} \; \big (\mathfrak {A} \nabla u_h,\nabla v_h)_{L^2(\Omega )} + \big ((\mathfrak {c}-z)u_h,v_h\big )_{L^2(\Omega )}= & {} \big (f,v_{h}\big )_{L^2(\Omega )} \\{} & {} \forall v_{h} \in \mathbb {V}_{h}. \end{aligned}$$

Given discretization parameters $\mathbb {V}_{h}\subseteq {\mathbb {H}}^{1}(\Omega )$, $\mathcal {N}_{\text {q}}\in \mathbb {N}$ and $k >0$, the fully discrete approximation to (2.4) is then given by

2.5 $$\begin{aligned} u_{h,k}:= \frac{1}{2\pi i} \sum _{j=-\mathcal {N}_{\text {q}}}^{\mathcal {N}_{\text {q}}} { \big (\psi _{\sigma ,\theta }(jk)\big )^{-\beta } \psi _{\sigma ,\theta }'(jk) \,R_h\big (\psi _{\sigma ,\theta }(jk)\big )f. } \end{aligned}$$

In order to keep presentation to a reasonable length, we focus on the spatially continuous setting. We only remark that discretization in space can be easily incorporated into the analysis. For low order finite elements one can follow [3]; for an exponentially convergent *hp*-FEM scheme we refer to [26]. $\square$

#### Remark 2.4

We should point out that for the elliptic problem, there exist methods based on the Balakrishnan formula (see also Sect. 4) which do not require complex arithmetic. On the other hand, since we are only approximating real valued functions, we can exploit the symmetry of (2.2) to only solve for $j \ge 0$, thus halving the number of linear systems. This results in (roughly) comparable computational effort for both the Balakrishnan and the double exponential schemes. Due to their better convergence the DE-schemes might therefore still be advantageous. $\square$

The convergence of the new method can be summarized in the following two theorems.

#### Theorem 2.5

Let *u* be the exact solution to (2.4) and assume $f \in {\mathbb {H}}^{2\rho }(\Omega )$ for some $\rho \ge 0$. Let $\beta \ge \overline{\beta }$ with $\overline{\beta } \in (0,1]$ and $u_k:=Q^{\mathcal {L}}(z^{-\beta },\mathcal {N}_{\text {q}}) f$ denote the approximation computed using stepsize $k>0$ and $\mathcal {N}_{\text {q}}\in \mathbb {N}$ quadrature points. Then, the following estimate holds for all $\varepsilon \ge 0$ and $r \in [0,\overline{\beta }/2]$:

$$\begin{aligned} \left\| u-u_{k}\right\| _{{\mathbb {H}}^{2r}}&= \left\| E^{\mathcal {L}}(z^{-\beta },\mathcal {N}_{\text {q}})f\right\| _{{\mathbb {H}}^{2r}} \\&\lesssim e^{-\frac{[p(\sigma ,\theta )-\varepsilon ] \sqrt{\beta +\rho -r}}{\sqrt{k}}}\left\| f\right\| _{{\mathbb {H}}^{2\rho }} \!+\! \Big [\exp \big (-\frac{\gamma }{k}\big ) + \exp \big (- \gamma e^{k \mathcal {N}_{\text {q}}}\big )\Big ] \left\| f\right\| _{{\mathcal {X}}}, \end{aligned}$$

where the rate $p(\sigma ,\theta )$ is given by

2.6 $$\begin{aligned} p(\sigma ,\theta ):= {\left\{ \begin{array}{ll} 2 \sqrt{2\pi \tan ^{-1}(\theta )} &{} \quad \text {for }\sigma =1, \\ 2\pi , &{} \quad \text {for }\sigma =1/2. \end{array}\right. } \end{aligned}$$

For $\varepsilon >0$, the implied constant and $\gamma$ may depend on $\varepsilon , r$, the smallest eigenvalue $\lambda _0$ of $\mathcal {L}$, $\overline{\beta }$, $\kappa$, $\theta$ and $\sigma$. But they are independent of $\rho$, $\beta$, *k*, and *f*. If $\varepsilon =0$, the constants may in addition depend on $\rho$ and $\beta$.

#### Remark 2.6

When comparing Theorem 2.5 to the estimates of the standard $\textrm{sinc}$-quadrature one might think that the double exponential method is inferior due to the $\sqrt{k}$ vs $k$ behavior. This misconception can be cleared up by considering the better decay properties of the double-exponential formula. It allows to choose $k\sim \ln (\mathcal {N}_{\text {q}})/\mathcal {N}_{\text {q}}$ compared to the standard $\textrm{sinc}$-quadrature choice of $k\sim \mathcal {N}_{\text {q}}^{-1/2}$ without the cutoff error becoming dominant. Using this choice, the exponential term scales like $\sqrt{\mathcal {N}_{\text {q}}/\ln (\mathcal {N}_{\text {q}})}$ for double exponential and $\sqrt{\mathcal {N}_{\text {q}}}$ for standard sinc respectively. As is shown in Sect. 4, the better constants in the exponential still often outweigh the presence of the $\ln$-term for the double-exponential quadrature. $\square$

#### Remark 2.7

For most of the computation, the convergence rate is determined by the factor $p(\sigma ,\theta )$ in Corollary 3.8. We observe that for $\theta =1$, picking $\sigma =1/2$ roughly doubles the convergence rate. Similarly, it often appears beneficial to pick larger values of $\theta$. Especially for $\sigma =1$, we get an asymptotic rate for $\theta \rightarrow \infty$, which is the same as in the case of $\sigma =1/2$. But we need to point out that increasing $\theta$ means that we have to decrease the value $d(\theta )$, which determines the rate in the higher orders terms of the form $e^{-\gamma /k}$, thus leading to those terms dominating in a larger and larger preasymptotic regime. Overall, the method using $\sigma =1/2$ and setting $\theta$ moderately large is expected to give the best convergence rates; cf. Sect. 4. $\square$

The previous theorem shows that in general, the convergence behaves like $\mathcal {O}(e^{-\frac{\gamma }{\sqrt{k}}})$. It also shows that, if the function *f* in the right-hand side has some additional smoothness, the method automatically detects this and delivers an improved convergence rate. If the additional smoothness is in the right Gevrey type classes, we can establish convergence which is beyond the root exponential behavior. The details can be found in the following theorem:

#### Theorem 2.8

Let *u* be the exact solution to (2.4) and assume that there exist constants $C_{f}, \omega , R_f>0$ such that $f \in \mathcal {G}^{\mathcal {L}}(C_f,R_f,\omega )$, i.e.,

$$\begin{aligned} \left\| f\right\| _{{\mathbb {H}}^{\rho }}&\le C_{f} \, R_f^\rho \,\big (\Gamma (\rho +1)\big )^{\omega } < \infty \qquad \forall \rho \ge 0. \end{aligned}$$

Assume that $\beta > \overline{\beta }$ with $\overline{\beta } \in (0,1]$. Let $u_k:=Q^{\mathcal {L}}(z^{-\beta },\mathcal {N}_{\text {q}})f$ denote the approximation computed using stepsize $k \in (0,1/2)$ and $\mathcal {N}_{\text {q}}\in \mathbb {N}$ quadrature points. Then, the following estimate holds:

$$\begin{aligned}&\left\| u-u_{k}\right\| _{\mathbb {H}^{\overline{\beta }}} =\left\| E^{\mathcal {L}}(z^{-\beta },\mathcal {N}_{\text {q}})\right\| _{\mathbb {H}^{\overline{\beta }}} \lesssim C_f \exp \Big (-\frac{\gamma }{k\left| \ln (k)\right| }\Big ) + C_f\exp \Big (- \gamma e^{k \mathcal {N}_{\text {q}}}\Big ) . \end{aligned}$$

The implied constant and $\gamma$ may depend on $\omega$, the smallest eigenvalue $\lambda _0$ of $\mathcal {L}$, $\kappa$, $\theta$, $\sigma$, $R_f$, $\overline{\beta }$, and $\omega$. If $\omega =0$, the logarithmic term may be removed.

### The parabolic problem

The second model problem we consider is a time-dependent fractional diffusion problem of parabolic type. We fix $\alpha , \beta \in (0,1]$ and a final time $T>0$. Given an initial condition $u_0\in {\mathcal {X}}$ and right-hand side $f \in C([0,T],{\mathcal {X}})$ we seek $u:[0,T]\rightarrow {\text {dom}}(\mathcal {L}^{\beta })$ satisfying

2.7 $$\begin{aligned} \partial ^{\alpha }_t u + \mathcal {L}^{\beta } u&= f \;\, \text {in } [0,T], \qquad u(t) \in {\text {dom}}(\mathcal {L}^{\beta }) \;\,\forall t>0, \quad \text {and}\quad u(0)=u_0, \end{aligned}$$

where $\partial _t^{\alpha }$ denotes the Caputo fractional derivative. Following [4], the solution *u* can be written using the Mittag–Leffler function $e_{\alpha ,\mu }$ (see (3.18)) as

2.8 $$\begin{aligned} u(t)&:= e_{\alpha ,1}\big (-t^{\alpha } \mathcal {L}^{\beta }\big ) u_0 + \int _{0}^{t} { \tau ^{\alpha -1} e_{\alpha ,\alpha }\big (-\tau ^{\alpha } \mathcal {L}^\beta \big ) f(t-\tau )\,d\tau }. \end{aligned}$$

Here we again use either the spectral or, equivalently, the Riesz–Dunford calculus to define the operators. We discretize this problem by using our double exponential formula. Namely for $$k >0 $$ and using $$\mathcal {N}_{\text {q}}\in \mathbb {N}$$ quadrature points,

2.9 $$\begin{aligned} u^k(t)&:=Q^{\mathcal {L}}\Big (e_{\alpha ,1}(-t^{\alpha } z^{\beta }),\mathcal {N}_{\text {q}}\Big ) u_0 + \int _{0}^{t}{\tau ^{\alpha -1} Q^{\mathcal {L}}\Big ( e_{\alpha ,\alpha }(-\tau ^{\alpha } z^{\beta }), \mathcal {N}_{\text {q}}\Big )\,f(t-\tau )d\tau }. \end{aligned}$$

#### Remark 2.9

In practice, in order to get a fully computable discrete scheme, one would again replace the resolvent by a Galerkin solver and the convolution in time by an appropriate numerical quadrature. For example, [4] presents a low order approximation scheme. In order to retain exponential convergence, [26] uses a scheme based on *hp*-FEM and *hp*-quadrature. We summarize the construction briefly. For a given degree $p \in \mathbb {N}_0$, and interval *I*, we denote the Gauss quadrature points and weights on $(-1,1)$ by $(x^{I,p}_j, w^{I,p}_j) \in I \times \mathbb {R}_+$, $j=0,\dots ,p$. See [11, Section 2.7] for details. We then consider a geometric mesh on (0, 1) with grading factor $\sigma \in (0,1)$ and parameter $L \in \mathbb {N}$, $L\le p$ given by

$$\begin{aligned} K_0:=(0, \sigma ^L),\, K_1:=(\sigma ^{L}, \sigma ^{L-1}),\, \dots ,\, K_{L}:=(\sigma ,1). \end{aligned}$$

On each one of these elements, we apply a Gauss quadrature, reducing the order as we approach the singularity, i.e., we get the nodes and weights as

$$\begin{aligned} (X,W):=\bigcup _{\ell =0}^{L}{\big \{(x^{K_\ell ,p-L+\ell }_j,w_j^{K_\ell ,p-L+\ell }) : j=0,\dots ,p \big \}}. \end{aligned}$$

The convolution in (2.7) is then replaced by

$$\begin{aligned} \int _{0}^{t}{\tau ^{\alpha -1} e_{\alpha ,\alpha }(-\tau ^{\alpha } z^{\beta }) \,d\tau }&\approx t\!\!\!\!\!\sum _{(x_j,w_j) \in (X,W)}{\!\!\!\!\!w_j (t\,x_j)^{\alpha -1} e_{\alpha ,\alpha } (-(t\,x_j)^{\alpha } z^{\beta }) f(t-(t x_j))\,}\\&=:h_t(z). \end{aligned}$$

In order to get a fully discrete scheme, this function is then discretized using the double exponential quadrature scheme:

$$\begin{aligned} \int _{0}^{t}{\tau ^{\alpha -1} e_{\alpha ,\alpha }(-\tau ^{\alpha } \mathcal {L}^{\beta })\,f(t-\tau )d\tau } \approx Q^{\mathcal {L}}( h_t, \mathcal {N}_{\text {q}}). \end{aligned}$$

In order to not overwhelm the presentation of the paper, we do not consider these types of discretization errors. The analysis of such errors could be taken almost verbatim from the references [3, 26]. $\square$

The analysis of the method again comes in the form of two theorems, one for the case of finite regularity and one for regularity in the Gevrey-type classes $\mathcal {G}^{\mathcal {L}}(C_f,R_f,\omega )$.

#### Theorem 2.10

Assume that either $\alpha +\beta <2$ or $\sigma =1$ (i.e., the case $\alpha =\beta =1$ and $\sigma =1/2$ is not allowed). Let *u* be the solution to (2.7). Assume $u_0 \in {\mathbb {H}}^{2\rho }$ for some $\rho > 0$, and $f \in C^{m}([0,T], {\mathbb {H}}^{2\rho })$ for some $m\in \mathbb {N}$. Let $u_k$ be the corresponding discretization using stepsize $k>0$ and $\mathcal {N}_{\text {q}}\in \mathbb {N}$ quadrature points as defined in (2.9).

Then, the following estimate holds for all $t \in (0,T)$, $r\in [0,\beta /2]$ and any $q<1$:

$$\begin{aligned} \left\| u(t)-u_{k}(t)\right\| _{{\mathbb {H}}^{2r}}\lesssim & {} \max \big (t^{-m-q\alpha },T^{\alpha } \big ) \Big (\left\| u_0\right\| _{{\mathbb {H}}^{2\rho }} + \sum _{j=0}^{m}{\max _{\tau \le t}\Vert {f^{(j)}(\tau )}\Vert _{{\mathbb {H}}^{2\rho }}}\Big ) \\{} & {} \times \Big ( e^{-\frac{\min \big \{p(\sigma ,\theta ) \sqrt{\beta +\rho -r},\gamma _1\sqrt{m/\alpha +q}\big \}}{\sqrt{k}}} + e^{-\gamma /k} + e^{-\gamma e^{k \mathcal {N}_{\text {q}}}}\Big ) , \end{aligned}$$

where $p(\sigma ,\theta )$ is as in (2.6) and $\gamma _1$ is the constant from Corollary 3.5. The implied constant and $\gamma$ may depend on *q*, *r*, the smallest eigenvalue $\lambda _0$ of $\mathcal {L}$, $\beta$, $\alpha$, $\kappa$, $\theta$, $\sigma$ and $\rho$.

#### Theorem 2.11

Assume that either $\alpha +\beta <2$ or $\sigma =1$ (i.e., the case $\alpha =\beta =1$ and $\sigma =1/2$ is not allowed). Let *u* be the solution to (2.7), and assume that the data satisfy

2.10 $$\begin{aligned} \begin{aligned} \left\| u_0\right\| _{{\mathbb {H}}^{\rho }}&\le C_{u_0} \, R_{u_0}^{\rho } \,\big (\Gamma (\rho +1)\big )^{\omega }< \infty \;\;\qquad \qquad \forall \rho \ge 0 \\ \big \Vert {f^{(n)}(t)}\big \Vert _{{\mathbb {H}}^{\rho }}&\le C_{f} \; R_{f}^{\rho +n} \,\big (\Gamma (\rho +1)\big )^{\omega } \big (n!\big )^{\omega } < \infty \quad \forall t \in [0,T],\; \forall \rho \ge 0,\;\forall n \in \mathbb {N}_0 . \end{aligned} \end{aligned}$$

Let $u_k$ be the corresponding discretization using stepsize $k \in (0,1/2)$ and $\mathcal {N}_{\text {q}}\in \mathbb {N}$ quadrature points as defined in (2.9). Then, the following estimate holds:

$$\begin{aligned} \left\| u(t)-u_{k}(t)\right\| _{{\mathbb {H}}^{\beta }}&\lesssim (1+t)\exp \Big (-\frac{\gamma }{k\left| \ln {t_\star }\right| \left| \ln {k}\right| }\Big ) + \big (t+ t^{-\gamma /2}\big )\exp \big (- \gamma e^{k \mathcal {N}_{\text {q}}}\big ) \end{aligned}$$

with $t_\star :=\min (t,1/2)$. The implied constant and $\gamma$ may depend on the smallest eigenvalue $\lambda _0$ of $\mathcal {L}$, $\beta$, $\theta$, $\sigma$ and the constants from (2.10).

## Error analysis

In this section, we analyze the quadrature error when applying a double exponential formula for discretizing certain integrals.

For $\theta \ge 1$, $\delta >0$ we define the sets

3.1 $$\begin{aligned} D_{d(\theta )}:=\Big \{z \in \mathbb {C}: \left| {\text {Im}}(z)\right|< d(\theta ) \Big \}, \; \text {and} \; D^{\textrm{exp}}_\delta :=\Big \{z \in \mathbb {C}: \left| {\text {Im}}(z)\right| < \delta e^{-\left| {\text {Re}}(z)\right| } \Big \}, \end{aligned}$$

where for each $\theta$, $d(\theta )$ is a constant which is assumed sufficiently small in order for Lemmas A.3, A.4, and A.8 to hold.

Since all the proofs analyzing the properties of $\psi _{\sigma ,\theta }$ are elementary but somewhat lengthy and cumbersome, they have been relegated to Appendix A. The most important properties are, that $y\mapsto \psi _{\sigma ,\theta }(y)$ for $y\in \mathbb {R}$ traces the contour in the definition of the Riesz Dunford calculus (see Definition 1.5), and that it is analytic in $D_{d(\theta )}$. The other important results concern the points where $\psi _{\sigma ,\theta }$ crosses the real axis, as these points correspond to (possible) poles in the integrand of Definition 1.5. The location of these points, as well as other important estimates are collected in Lemma A.8. Roughly summarizing, the finitely many points *y* satisfying $\psi _{\sigma ,\theta }(y)=\lambda$ have distance $1/\ln (\lambda )$ from the real axis. Away from such points $\left| \psi _{\sigma ,\theta }(y)-\lambda \right| > rsim \lambda$ holds and for $y \rightarrow \pm \infty$ the function $\psi _{\sigma ,\theta }$ behaves doubly-exponential (Lemma A.4).

### Abstract analysis of ${\text {sinc}}$-quadrature

In this section, we collect some results on $\textrm{sinc}$-quadrature formulas.

#### Remark 3.1

As is common in the literature, we define the $\textrm{sinc}$ function as

$$\begin{aligned} \textrm{sinc}(\zeta ):={\left\{ \begin{array}{ll} \frac{\sin (\pi \zeta )}{\pi \zeta } &{} \quad \zeta \ne 0 \\ 1 &{} \quad \zeta =0. \end{array}\right. } \end{aligned}$$

The following result is the main work-horse when analyzing ${\text {sinc}}$-quadrature schemes. In order to reduce the required notation, we use a simplified version of [32, Problem 3.2.6].

#### Proposition 3.2

(Bialecki, see [32, Problem 3.2.6 and Theorem 3.1.9]) We make the following assumptions on *g*:

(i) *g* is a meromorphic function on the infinite strip $D_{d(\theta )}$. It is also continuous on $\partial D_{d(\theta )}$. The poles $\big (p_{\ell }\big )_{\ell =1}^{N_p}$ are all simple and located in $D_{d(\theta )}\setminus \mathbb {R}$.
(ii) There exists a constant $C>0$ independent of $y \in \mathbb {R}$ such that for sufficiently large $y > 0$,
  3.2 $$\begin{aligned} \int _{-d(\theta )}^{d(\theta )}{\left| g(y+iw)\right| \,dw}&\le C. \end{aligned}$$
(iii) We have
  3.3 $$\begin{aligned} N(g,D_{d(\theta )}):=\int _{-\infty }^{\infty }{\left| g(y+id(\theta ))\right| + \left| g(y-id(\theta ))\right| \,dy} < \infty . \end{aligned}$$

Denote by ${\text {res}}(g;p_\ell )$ the residue of *g* at $p_\ell$, and define $\gamma (k;p_\ell ):=\frac{1}{\sin (\pi p_\ell /k)}.$

Then for all $k > 0$, using $s_\ell :={\text {sign}}({\text {Im}}(p_\ell ))$:

3.4 $$\begin{aligned}{} & {} \Big |{ \int _\mathbb {R}{g(t) \, dt} - k \sum _{n=-\infty }^{\infty }{g(k \, n)} - \pi \sum _{\ell =1}^{N_p} {e^{i\frac{s_\ell \pi p_\ell }{k}} {\text {res}}(g;p_\ell ) \gamma (k;p_\ell ) } }\Big | \nonumber \\{} & {} \quad \le \frac{e^{-2\pi d(\theta )/k}}{1-e^{-2\pi d(\theta )/k}} N(g,D_{d(\theta )}). \end{aligned}$$

Proposition 3.2 requires certain decay properties for the integrand in a complex strip, and thus is not always applicable. As is shown in Appendix A, the transformation $\psi _{\sigma ,\theta }$ maps partly into the left-half plane. One can even show that the real part changes sign infinitely many times when evaluating along a line of fixed imaginary part. If we therefore consider the case when $f(z):=e^{-z}$ is the exponential function, this means that $f \circ \psi$ is exponentially increasing in such regions. This puts showing estimates of the form required in Proposition 3.2 (iii) out of reach.

On the other hand, Lemma A.5 shows that for $\sigma =1$, restricted to the domain $D^{\textrm{exp}}_\delta$, the map $\psi _{\sigma ,\theta }$ stays in the right half-plane. Here the exponential function is decreasing. Similarly, the Mittag–Leffler function $e_{\alpha ,\mu }$ is decreasing on slightly larger sectors, allowing for the choice of $\sigma =1/2$ if $\alpha < 1$. This motivates the following modification of Proposition 3.2.

#### Lemma 3.3

Assume that $g: D^{\textrm{exp}}_\delta \rightarrow \mathbb {C}$ is holomorphic and is doubly-exponentially decreasing, i.e., there exist constants $C_g > 0$, $\mu _g >0,$ such that *g* satisfies

3.5 $$\begin{aligned} \left| g(y)\right| \le C_g \exp \big (-\mu _g e^{{\text {Re}}(y)}\big ) \qquad \forall y \in D^{\textrm{exp}}_\delta . \end{aligned}$$

Then, for all , there exists a constant $C>0$ which is independent of *k*, $\mu$ and *g* such that the following error estimate holds:

3.6 $$\begin{aligned} \left| \int _{\mathbb {R}}{g(t) dt} - k\sum _{n=-\infty }^{\infty }{g(k\,n)}\right|&\le C C_g k\,\exp \Big ({- \sqrt{8\pi \delta } \,\frac{\sqrt{\mu _g-2\varepsilon }}{\sqrt{k}}}\,\Big ). \end{aligned}$$

#### Proof

We closely follow the proof of [21, Theorem 2.13], but picking a different contour and later exploiting the strong decay properties of *g*.

For $N \in \mathbb {N}$, set $R_N:=\Big \{ y \in \mathbb {C}: \left| {\text {Re}}(y)\right| \le (N+\frac{1}{2}), \left| {\text {Im}}(y)\right| \lesssim \delta \, e^{-\left| {\text {Re}}(y)\right| }\Big \}$. For fixed $t \in \mathbb {R}$, we fix *N* large enough such that $t \in R_N$. By applying the residue theorem to the function

$$\begin{aligned} h(y):=\frac{\sin (\pi \, t / k) g(y)}{(t-y) \sin (\pi y/k)}, \end{aligned}$$

one can show the equality

$$\begin{aligned} g(t) - k\sum _{n=-N}^{N}{g(n\,k) \textrm{sinc}\Big (\frac{t-nk}{k}\Big )}&= \int _{\partial R_N}{ \frac{\sin (\pi \, t / k)g(y)}{(t-y) \sin (\pi y/k)} \,dy }. \end{aligned}$$

Since asymptotically *g*(*t*) decreases doubly exponentially, while $1/\sin (\pi y/k)$ only grows exponentially along the path $\{(\xi ,\delta \, e^{-\xi }), \, \xi \in \mathbb {R}\}$, we can pass to the limit $N \rightarrow \infty$ to get the representation

3.7 $$\begin{aligned} g(t) - k\sum _{n=-\infty }^{\infty }{g(k\,n) \textrm{sinc}\Big (\frac{t-nk}{k}\Big )}&= \int _{\partial D^{\textrm{exp}}_\delta }{ \frac{\sin (\pi \, t / k)g(y)}{(t-y) \sin (\pi y/k)} \,dy }. \end{aligned}$$

Integrating (3.7) over $\mathbb {R}$ and exchanging the order of integration gives:

3.8 $$\begin{aligned} \int _{\mathbb {R}}{g(t) \, d\tau } - k\sum _{n=-\infty }^{\infty }{g(k\,n)}&= \int _{\partial D^{\textrm{exp}}_\delta }{ \frac{g(y)}{ \sin (\pi y/k)} \int _{\mathbb {R}}{ \frac{\sin (\pi \, t / k)}{t-y} \, dt} \, dy.} \nonumber \\&= \pi \int _{\partial D^{\textrm{exp}}_\delta }{ \frac{g(y)}{ \sin (\pi y/k)} e^{\frac{i{\text {sign}}({\text {Im}}(y)) \pi y}{k}} \, dy} , \end{aligned}$$

where in the last step we invoked [21, Lemma 2.19] to explicitly evaluate the integral. What remains to be done is bound the integral on the right-hand side. For simplicity, we focus on the upper-right half-plane. The other cases follow analogously. There, we can parameterize $\partial D^{\textrm{exp}}_\delta$ as $y=\xi + i\delta \, e^{-\xi }$. We estimate

3.9 $$\begin{aligned} \left| \frac{g(y)}{ \sin (\pi y/k)} e^{\frac{i{\text {sign}}{{\text {Im}}(y)} \pi y}{k}}\right|&=\; |g(y)| \frac{|e^{\frac{i\pi y}{k}}|}{{|e^{i\pi y/k} - e^{-i\pi y/k}|}} \nonumber \\&= \; \left| g(y)\right| \frac{\exp \Big ({-\pi \delta \,\frac{ e^{-\xi }}{k}}\Big )}{\exp \Big ({\pi \delta \,\frac{ e^{-\xi }}{k}}\Big )- \exp \Big ({-\pi \delta \,\frac{ e^{-\xi }}{k}}\Big )} \nonumber \\&=\; \left| g(y)\right| \frac{\exp \Big ({-2\pi \delta \,\frac{ e^{-\xi }}{k}}\Big )}{1- \exp \Big ({-2\pi \delta \,\frac{ e^{-\xi }}{k}}\Big )}\nonumber \\ {}&\lesssim \; \left| g(y)\right| k\,e^{\xi } \exp \Big ({-2\pi \delta \,\frac{ e^{-\xi }}{k}}\Big ) \nonumber \\&{\mathop {\lesssim }\limits ^{{(3.5)}}} \; C_g k\exp \Big (-\mu _g e^\xi + \xi - \frac{2\pi \, \delta e^{-\xi }}{k} \Big ) \end{aligned}$$

For $\varepsilon > 0$, we can absorb the linear $\xi$-term into the first exponential, and estimate:

$$\begin{aligned} (3.9)&\lesssim \varepsilon ^{-1} C_g k\exp \Big (-(\mu _g-2\varepsilon ) e^\xi - \frac{2\pi \, \delta e^{-\xi }}{k} \Big ) \exp \Big ( -\varepsilon e^\xi \Big ) \end{aligned}$$

where the second term will be used to regain integrability, whereas the first one will give us approximation quality. For $\xi = 0$ and $\xi \rightarrow \infty$, we get sufficient bounds to prove (3.6). We thus have to look for maxima of the function with respect to $\xi$ in between $(0,\infty )$. Due to monotonicity of the exponential, we focus on the argument and set $\tau :=e^{\xi }.$ By setting its derivative to zero we get that the map

$$\begin{aligned} \tau \mapsto - (\mu _g - 2\varepsilon ) \tau - \frac{2\pi \, \delta }{\tau \, k} \quad \text { is maximized for } \quad \tau _{\max } = \sqrt{\frac{2\delta \pi }{ k (\mu _g - 2\varepsilon )}}. \end{aligned}$$

Inserting all this into (3.8), we get

$$\begin{aligned} \Big |{\int _{\mathbb {R}}{g(t) d\tau }-k\sum _{n=-\infty }^{\infty }{\!g(k\,n)}}\Big |&\lesssim C_g k \exp \Big (\!\!-\!\!\sqrt{8\pi \delta }\sqrt{\frac{\mu _g - 2 \varepsilon }{k}}\Big ) \!\!\int _{0}^\infty { \!\!\!\! \exp (-\varepsilon e^{\left| {\text {Re}}(y)\right| }) \, d\xi }\\&\lesssim C_g k \exp \Big (- 2 \sqrt{2\pi \delta }\sqrt{\frac{\mu _g - 2 \varepsilon }{k}}\Big ). \end{aligned}$$

$$\square $$

#### Remark 3.4

It is also possible to admit meromorphic functions with finitely many poles into Lemma 3.3, as long as additional error terms analogous to (3.4) are introduced. Since we will not need this generalization we stay in the analytic setting. $\square$

While Lemma 3.3 provides a reduced rate of convergence compared to the more-standard $\textrm{sinc}$-quadrature of Proposition 3.2 ($k^{-1/2}$ vs $k^{-1}$), thus removing the advantage we want to achieve by using the double exponential transformation, we will later consider a class of functions which decay fast enough to allow us to tune the parameter $\mu \sim k^{-1}$ to regain almost full speed of convergence.

Finally, we show how the transformation $\psi _{\sigma ,\theta }$ and the operator $\mathcal {L}$ enter the estimates. The next corollary also showcases how the cutoff error is controlled.

#### Corollary 3.5

Let ${\mathcal {O}} \subseteq \mathbb {C}$ contain the right half-plane, and if $\sigma =1/2$ also a sector

$$\begin{aligned} S_{\omega }:=\big \{z \in \mathbb {C}: \left| {\text {Arg}}(z)\right| \le \omega \big \} \qquad \text {for some} \quad \omega >\frac{\pi }{2}. \end{aligned}$$

Assume that $g: {\mathcal {O}} \rightarrow \mathbb {C}$ is analytic and satisfies the polynomial bound

$$\begin{aligned} \left| g(z)\right| \le C_{g} (1+\left| z\right| )^{-\mu } \qquad \text {for}\, \mu \in \mathbb {R}. \end{aligned}$$

Then, for all $\varepsilon > 0$, $s, r \in \mathbb {R}$ such that $\mu - r + s -2\varepsilon >0$, the quadrature errors can be bounded by:

$$\begin{aligned} \left\| E^{\mathcal {L}}(g,\mathcal {N}_{\text {q}})\right\| _{{\mathbb {H}}^{2s} \rightarrow {\mathbb {H}}^{2r}}&\le C\, C_g \Big [e^{- \hat{\gamma } \frac{ \sqrt{\mu - r +s -2\varepsilon }}{\sqrt{k}}} +\exp \big ({- (\mu -r+s) \gamma e^{k \mathcal {N}_{\text {q}}}}\big )\Big ]. \end{aligned}$$

The constant *C* is independent of *g*, *k*,*r*,*s* and $\beta$, but may depend on $\varepsilon$, $\sigma$, $\theta$. The rate $\hat{\gamma }$ depends on $\theta$ and $\omega$. $\gamma$ depends on $\sigma$.

#### Proof

Let $(\lambda _j, v_j)_{j=0}^{\infty }$ denote the eigenvalues and eigenfunctions of the self-adjoint operator $\mathcal {L}$. Following [3], plugging the eigen-decomposition of a function *u* into the Riesz–Dunford calculus, we can write the exact function $g(\mathcal {L}) u$ as

$$\begin{aligned} g(\mathcal {L}) u&=\sum _{j=0}^{\infty }{\Big (\frac{1}{2\pi i}\int _{\mathcal {C}}{g(\psi _{\sigma ,\theta }(y)) (\psi _{\sigma ,\theta }-\lambda _j)^{-1} \psi _{\sigma ,\theta }'(y)\big (u,v_j\big )_{L^2(\Omega )} \,dy} \Big )\;v_j} \end{aligned}$$

and analogously for the discrete approximation $Q^{\mathcal {L}}(g,\mathcal {N}_{\text {q}})u$. For the norm, as defined in (1.1), this means:

$$\begin{aligned} \left\| E^{\mathcal {L}}(g,\mathcal {N}_{\text {q}})\right\| ^2_{{\mathbb {H}}^{2r}}&= \frac{1}{4\pi ^2} \sum _{j=0}^{\infty }{\Big |(1+\lambda _j^{r}) E^{\lambda _j}(g,\mathcal {N}_{\text {q}})\big (u,v_j\big )_{L^2(\Omega )}\Big |^2} \\&\lesssim \sup _{\lambda \ge \lambda _0} \big |(1+\lambda ^{r-s}) E^{\lambda }(g,\mathcal {N}_{\text {q}})\big |^2 \left\| u\right\| ^2_{{\mathbb {H}}^{2s}}. \end{aligned}$$

We have thus reduced the problem to one of scalar quadrature, for which we aim to apply Lemma 3.3. We fix $\lambda> \lambda _0>\kappa$. $\psi _{\sigma ,\theta }$ maps $D^{\textrm{exp}}_\delta$ analytically to ${\mathcal {O}}$ via Lemma A.5 ($\delta$ depends on $\theta$ and $\omega$). What remains to be shown is a pointwise bound for the function

$$\begin{aligned} h_\lambda (y):=\lambda ^{r-s}g(\psi _{\sigma ,\theta }(y))(\psi _{\sigma ,\theta }-\lambda )^{-1} \psi _{\sigma ,\theta }'(y) \qquad \forall y \in D^{\textrm{exp}}_\delta . \end{aligned}$$

By distinguishing the cases $\left| \psi _{\sigma ,\theta }(y)\right| <\lambda /2$ and $\left| \psi _{\sigma ,\theta }(y)\right| \ge \lambda /2$ we get using either (A6) or Lemma A.5

$$\begin{aligned} \lambda \left| \psi _{\sigma ,\theta }(y)-\lambda \right| ^{-1} \left| \psi _{\sigma ,\theta }'(y)\right| \lesssim \left| \psi _{\sigma ,\theta }(y)\right| \cosh ({\text {Re}}(y)). \end{aligned}$$

We conclude using Lemma A.5:

$$\begin{aligned} \left| h_\lambda (y)\right|&\le C_g \left| \psi _{\sigma ,\theta }(y)\right| ^{-\mu } \big (\lambda \left| \psi _{\sigma ,\theta }(y)-\lambda \right| ^{-1} \left| \psi _{\sigma ,\theta }'(y)\right| \big )^{r-s} \\&\times \big (\left| \psi _{\sigma ,\theta }(y)-\lambda \right| ^{-1} \left| \psi _{\sigma ,\theta }'(y)\right| \big )^{1-r+s} \\&\lesssim C_g \left| \psi _{\sigma ,\theta }(y)\right| ^{-\mu +r - s} \cosh ({\text {Re}}(y)). \end{aligned}$$

The double exponential growth of $\psi _{\sigma ,\theta }$ (see Lemma A.4) then gives after absorbing the $\cosh$ term by slightly adjusting $\varepsilon$:

3.10 $$\begin{aligned} \left| h_\lambda (y)\right|&\le C_g c_1 \exp \big (-\gamma _1(\mu -r + s)e^{|{\text {Re}}(y)|}\big ) \cosh ({\text {Re}}(y)) \nonumber \\&\lesssim C_g \exp \big (-\gamma _1(\mu -r + s-\varepsilon )e^{|{\text {Re}}(y)|}\big ). \end{aligned}$$

Using Lemma 3.3, with $\mu _g:=\gamma _1(\mu -r + s-\varepsilon )$ then gives, after readjusting $\varepsilon$:

$$\begin{aligned} \lambda ^{r-s}\big |{E^{\lambda }(g)}\big |&=\frac{1}{2\pi }\Big | \int _{\mathbb {R}}{h_{\lambda }(y)\,dy} - k\sum _{n=-\infty }^{\infty }{h_\lambda (k\,n)} \Big | \lesssim C_g e^{-\widehat{\gamma } \frac{\sqrt{\mu - r+s -2\varepsilon }}{\sqrt{k}}} \end{aligned}$$

with $\hat{\gamma }:=\sqrt{8\pi \delta \gamma _1}$. The cutoff error is handled easily, also using the estimate (3.10). We calculate

$$\begin{aligned} \left| Q^{\lambda }(g)-Q^{\lambda }(g,\mathcal {N}_{\text {q}})\right|&\le k \!\!\! \sum _{ \left| j\right| \ge \mathcal {N}_{\text {q}}+1}{\!\!\!\left| h_\lambda (\psi _{\sigma ,\theta }(j\,k))\right| } \\&\le C_g \,k \lambda ^{-r+s} \!\!\! \sum _{ \left| j\right| \ge \mathcal {N}_{\text {q}}+1}{\!\!\!\exp \big (-\gamma _1(\mu -r+s) e^{jk}\big )} \\&\lesssim C_g \lambda ^{-r+s} \exp \big (-\gamma _1 (\mu -r+s) e^{ \mathcal {N}_{\text {q}}k}\big ), \end{aligned}$$

where the last step follows by estimating the sum by the integral and elementary estimates. $\square$

###  The elliptic problem

In this section, we analyze the error when discretizing the elliptic fractional diffusion problem from Sect. 2.1. In order to analyze the quadrature error, we need to understand a specific scalar function. This is done in the next Lemma.

#### Lemma 3.6

Fix $\lambda> \lambda _0 > \kappa$ and $\beta > 0$. For $y \in \mathbb {R}$, define the function

$$\begin{aligned} g_\lambda ^\beta (y)&:=\big (\psi _{\sigma ,\theta }(y)\big )^{-\beta } \Big (\psi _{\sigma ,\theta }(y)-\lambda \Big )^{-1} \psi _{\sigma ,\theta }'(y). \end{aligned}$$

Then the following statements hold:

(i) $g_\lambda ^{\beta }$ can be extended to a meromorphic function on $D_{d(\theta )}$. It has finitely many poles. All poles *p* satisfy $\psi _{\sigma ,\theta }(p)=\lambda$ and are all simple. For any $\nu \ge 0$, the number of poles within the strip
  $$\begin{aligned} \nu -\frac{1}{\ln (\lambda /\kappa )}\le \left| {\text {Im}}(y)\right| \le \nu +\frac{1}{\ln (\lambda /\kappa )} \end{aligned}$$
  can be bounded independently of $\nu$, $\beta$ and $\lambda$. The imaginary part of *p* can be bounded away from zero and for large $\lambda$, the following asymptotics hold:
  3.11 $$\begin{aligned} \left| {\text {Im}}(p)\right|&\ge {\left\{ \begin{array}{ll} \frac{\tan ^{-1}(\theta )}{\ln (\lambda /\kappa )} - \mathcal {O}\Big (\frac{1}{\ln (\lambda /\kappa )^2}\Big ) &{} \quad \text {if}\, \sigma =1, \\ \frac{\pi }{2\ln (\lambda /\kappa )} - \mathcal {O}\Big (\frac{1}{\ln (\lambda /\kappa )^2}\Big ) &{} \quad \text {if}\, \sigma =1/2. \end{array}\right. } \end{aligned}$$
  where the implied constants depend on $\theta$, $\kappa$, and $\lambda _0$.
(ii) There exist constants $C>0$, $\gamma > 0$, independent of $\lambda$ and $\beta$ and a value $d_{\lambda } \in (d(\theta )/2,d(\theta ))$ such that $g_\lambda ^\beta$ satisfies the bounds
  3.12 $$\begin{aligned} \big |{(1+\lambda ^{\frac{\beta }{2}})g_\lambda ^\beta (a \pm i\,d_{\lambda } )}\big | \le C \exp \big (- \gamma \beta e^a\big ) \qquad \forall a \in \mathbb {R}. \end{aligned}$$
(iii) There exists a constant $C>0$ such that for $d_{\lambda }$ from (ii) and $\beta \ge \overline{\beta }$ with $\overline{\beta } \in (0,1]$
  $$\begin{aligned} \int _{\mathbb {R}} { (1+\lambda ^{\frac{\overline{\beta }}{2}})\big |{g_\lambda ^\beta (w\pm id_\lambda )}\big | \, dw}&\le C < \infty . \end{aligned}$$
  The constant *C* may depend on $\overline{\beta }$ but can be chosen independently of $\lambda$ and $\beta$.

#### Proof

*Proof of* (i): We note that by Lemma A.3, $\psi _{\sigma ,\theta }$ is non-vanishing in $D_{d(\theta )}$. Since $D_{d(\theta )}$ is simply connected, we may define

$$\begin{aligned} h(y):=\ln (\kappa )+\int _0^y{ \frac{\psi _{\sigma ,\theta }'(\zeta )}{\psi _{\sigma ,\theta }(\zeta )} \,d\zeta }. \end{aligned}$$

It is easy to check that on $\mathbb {R}$ we have $h(y)=\ln (\psi _{\sigma ,\theta }(y))$ since the derivative as well as the value at $y=0$ coincide. Thus, defining

$$\begin{aligned} g_\lambda ^\beta (y) :=e^{-\beta h(y)} \big (\psi _{\sigma ,\theta }(y)-\lambda \big )^{-1} \psi _{\sigma ,\theta }'(y) \end{aligned}$$

provides a valid meromorphic extension. The only poles are located where $\psi _{\sigma ,\theta }(z)=\lambda$. By Lemma A.8 (i), the number of such poles within strips of width $\ln (\lambda )^{-1}$ is uniformly bounded. By Lemma A.3, $\psi _{\sigma ,\theta }'$ has no zeros in the domain $D_{d(\theta )}$, which means all the poles are simple. The bound on the imaginary part follows from Lemma A.8 (ii).

*Proof of* (ii): We first note for $y=a \pm id_{\lambda }$, if $\lambda < \left| \psi _{\sigma ,\theta }(y)\right| /2$, the trivial estimate $\left| \psi _{\sigma ,\theta }(y) - \lambda \right| ^{-1} \le \frac{2}{\left| \psi (y)\right| }$ holds. Otherwise, we use Lemma A.8(iii) to get

$$\begin{aligned} \left| \psi _{\sigma ,\theta }(y) - \lambda \right| ^{-1} \lesssim \lambda ^{-1} \le 2\left| \psi _{\sigma ,\theta }(y)\right| ^{-1}. \end{aligned}$$

Overall, we can estimate using Lemma A.4

$$\begin{aligned} |{g^\beta _\lambda (y)}|&\lesssim \left| \psi _{\sigma ,\theta }(y)\right| ^{-\beta } \left| \psi _{\sigma ,\theta }(y)-\lambda \right| ^{-1} \left| \psi _{\sigma ,\theta }'(y)\right| \lesssim \left| \psi _{\sigma ,\theta }(y)\right| ^{-\beta -1} \left| \psi _{\sigma ,\theta }'(y)\right| \\&\lesssim \exp (-\gamma \beta e^{{\text {Re}}(y)}), \end{aligned}$$

where in the last step, we used that $\psi _{\sigma ,\theta }'$ has the same asymptotic behavior as $\psi _{\sigma ,\theta }$ up to single exponential terms, which we absorb into the double exponential by slightly reducing $\gamma$.

Looking at $|\lambda ^{\frac{\overline{\beta }}{2}} {g^\beta _\lambda (y)}|$, one can calculate using two different ways to estimate $\psi _{\sigma ,\theta }(y)-\lambda$:

$$\begin{aligned} \lambda ^{\overline{\beta }/2}|{g^\beta _\lambda (y)}|&\lesssim \left| \psi _{\sigma ,\theta }(y)\right| ^{-\beta } \!\big (\underbrace{\!\lambda \left| \psi _{\sigma ,\theta }(y)-\lambda \right| ^{-1}}_{\lesssim 1}\!\big )^{\beta /2} \!\big (\!\underbrace{\left| \psi _{\sigma ,\theta }(y)-\lambda \right| ^{-1}\!}_{\lesssim \left| \psi _{\sigma ,\theta }(y)\right| ^{-1}}\big )^{1-{\overline{\beta }/2}}\!\left| \psi _{\sigma ,\theta }'(y)\right| \\&\lesssim \left| \psi _{\sigma ,\theta }(y)\right| ^{-\beta } \left| \psi _{\sigma ,\theta }(y)\right| ^{-1+\beta /2} \left| \psi _{\sigma ,\theta }'(y)\right| \\&{\mathop {\lesssim }\limits ^{{Lemma~A.4}}} \qquad \exp \Big (-\frac{\gamma \beta }{2} e^{{\text {Re}}(y)}\Big ). \end{aligned}$$

The integral bound then follows easily from the pointwise ones. $\square$

#### Theorem 3.7

Fix $\lambda _0 > \kappa$, $\overline{\beta }\in (0,1]$ and $r \in [0,\overline{\beta }/2]$. Then there exist constants $C>0$, $\gamma>0, \gamma _1>0$ such that for $\lambda > \lambda _0$, $\beta \ge \overline{\beta }$, $k>0$, $\mathcal {N}_{\text {q}}\in \mathbb {N}$, the following estimate holds

3.13a $$\begin{aligned} \lambda ^{r}\left| E^{\lambda }(z^{-\beta },\mathcal {N}_{\text {q}})\right|&\lesssim k^2\max (1,\ln (\lambda ))^2 \lambda ^{-\beta +r} e^{-\frac{\max \{p(\sigma ,\theta ,\lambda ),\gamma _1\}}{k\max (1,\ln (\lambda /\kappa ))}} \nonumber \\&\quad \ +e^{-\frac{\gamma }{k}} + \exp \big (- \gamma \beta e^{k \mathcal {N}_{\text {q}}}\big ), \end{aligned}$$

where the rate is given by

3.13b $$\begin{aligned} p(\sigma ,\theta ,\lambda )= {\left\{ \begin{array}{ll} 2\pi \tan ^{-1}(\theta )- \frac{c_2}{\ln (\lambda /\kappa )} &{} \quad \text {if}\, \sigma =1, \\ \pi ^2 - \frac{c_2}{\ln (\lambda /\kappa )} &{} \quad \text {if }\, \sigma =1/2. \end{array}\right. } \end{aligned}$$

Thus for $k \sim \ln (\mathcal {N}_{\text {q}})/\mathcal {N}_{\text {q}}$ we get (almost) exponential convergence:

3.14 $$\begin{aligned} \lambda ^{r}\Big |E^{\lambda }(z^{-\beta },\mathcal {N}_{\text {q}})\Big |&\lesssim k^2 \max (1,\ln (\lambda /\kappa ))^2 \lambda ^{-\beta +r} e^{-\frac{\max \big \{p(\sigma ,\theta ,\lambda ),\gamma _1\big \}\mathcal {N}_{\text {q}}}{\ln (\lambda /\kappa )\ln (\mathcal {N}_{\text {q}})}} +e^{-\gamma ' \frac{\mathcal {N}_{\text {q}}}{\ln (\mathcal {N}_{\text {q}})}}. \end{aligned}$$

The implied constants and $\gamma$ may depend on $\lambda _0$, $\overline{\beta }$, $\sigma$, $\theta$ and $\kappa$.

#### Proof

To cut down on notation, we only consider the case $\ln (\lambda /\kappa )\ge c_1 >1$ so that the first term in the minimum of  (3.11) dominates. If $\lambda$ is small, the error can be absorbed into the $e^{-\gamma /k}$ term. The error $E^{\lambda }(z^{-\beta },\mathcal {N}_{\text {q}})$ corresponds to approximating $g_{\lambda }^{\beta }$ by $\textrm{sinc}$ quadrature. We split the error into two parts, the quadrature error and the cutoff error.

$$\begin{aligned}{} & {} \lambda ^r\Big |{\int _{-\infty }^{\infty }{g_{\lambda }^{\beta }(y) \, dy} - k \sum _{ j= -\mathcal {N}_{\text {q}}}^{\mathcal {N}_{\text {q}}} g_\lambda ^\beta (j\,k)}\Big | \\{} & {} \quad \le \underbrace{\lambda ^r\Big |{\int _{-\infty }^{\infty }{g_{\lambda }^{\beta }(y)\,dy} - k \sum _{ j= -\infty }^{\infty } g_{\lambda }^{\beta }(j\,k)}\Big |}_{=E^{\lambda }(z^{-\beta })} + \underbrace{k \lambda ^r \!\!\! \sum _{ \left| j\right| > \mathcal {N}_{\text {q}}+1} \left| g_{\lambda }^{\beta }(j\,k)\right| }_{ =:E_c}. \end{aligned}$$

The term $E_c$ can be handled by the same argument as in Corollary 3.5. We therefore focus on the quadrature error $E^{\lambda }(z^{-\beta })$ and apply Proposition 3.2. By Lemma 3.6(iii) it holds that $N\big (g_\lambda ^\beta ,D_{d(\theta )}\big ) < \infty$. To satisfy assumption (ii), it suffices that (for sufficiently large *y*) the vertical strips do not contain any poles and we can use the asymptotics of Lemma 3.6(ii).

By Lemma 3.6, there are at most finitely many simple poles. The residue of the function at these poles can be easily calculated using the well-known rule

$$\begin{aligned} {\text {res}}\big (f/g: z_0\big ) = \frac{f(z_0)}{g'(z_0)}, \end{aligned}$$

provided that *f* is analytic and $g'(z_0)\ne 0$. In our case this means, if $\psi _{\sigma ,\theta }(y_\lambda )=\lambda$:

$$\begin{aligned} {\text {res}}(g^{\beta }_ \lambda ; y_\lambda )&=\frac{e^{- \beta h(y_\lambda )}\psi '(y_ \lambda )}{\psi '(y_\lambda )} = e^{-2 i \pi \beta \zeta } (\psi (y_ \lambda ))^{-\beta } = e^{-2 i \pi \beta \zeta } \lambda ^{-\beta }, \end{aligned}$$

where $\zeta \in \mathbb {N}_0$ denotes the branch of the complex logarithm picked by *h*.

Thus, for a single pole $y_\lambda$ with $s_{y_\lambda }:={\text {sign}}({\text {Im}}(y_\lambda ))$, recalling the definition of , we can estimate

$$\begin{aligned} \left| e^{i\frac{\pi s_{y_\lambda } y_\lambda }{k}} \, {\text {res}}(g^\beta _\lambda ,y_\lambda ) \, \gamma (k;y_\lambda )\right|&\lesssim \lambda ^{-\beta }\Big | \frac{e^{i\pi s_{y_\lambda } y_{\lambda }/k}}{ e^{i\pi s_{y_\lambda } y_{\lambda }/k} - e^{-i\pi s_{y_\lambda } y_{\lambda }/k}}\Big | \\&= \lambda ^{-\beta } \frac{e^{-2\pi \left| {\text {Im}}(y_\lambda )\right| /k}}{1-e^{-2\pi \left| {\text {Im}}(y_\lambda )\right| /k}}. \end{aligned}$$

By Lemma 3.6(i), we can group poles into buckets of size $\frac{1}{\ln (\lambda /\kappa )}$, denoted by

$$\begin{aligned} B_{\ell }:=\Bigg \{ y: \psi _{\sigma ,\theta }(y) = \lambda \, \text {with} \, \frac{\frac{p(\sigma ,\theta ,\lambda )}{2\pi }+\ell }{\ln (\lambda /\kappa )}\! \le \!\left| {\text {Im}}(y)\right| \! \le \! \min \bigg (\frac{\frac{p(\sigma ,\theta ,\lambda )}{2\pi }+\ell +1}{\ln (\lambda /\kappa )},d(\theta )\bigg ) \Bigg \} \end{aligned}$$

such that the number of elements in each bucket $B_{\ell }$ is uniformly bounded (independently of $\lambda$, $\beta$ and $\ell$). This allows us to calculate for the pole contribution in Proposition 3.2:

$$\begin{aligned}{} & {} \Big |\pi \sum _{y_\lambda \in P^y_\lambda } {e^{i\frac{s_\lambda \pi y_\lambda }{k}} \, {\text {res}}(g_\lambda ^{\beta };y_\lambda ) \, \gamma (k;y_\lambda )}\Big | \\{} & {} \quad \le \lambda ^{-\beta } \pi \sum _{\ell =0}^{\infty } { \Big | \sum _{y_\lambda \in B_{\ell }} { e^{i\frac{ \pi s_{y_\lambda }y_\lambda }{k}} \, \gamma (k;y_\lambda )} \Big |} \lesssim \lambda ^{-\beta } \sum _{\ell =0}^{\infty } { \frac{e^{-\frac{p(\sigma ,\theta ,\lambda )+\ell }{k\ln (\lambda /\kappa )}}}{1-e^{-\frac{p(\sigma ,\theta ,\lambda )+\ell }{k\ln (\lambda /\kappa )}}}} \\{} & {} \quad \lesssim \frac{\lambda ^{-\beta } }{1-e^{-\frac{p(\sigma ,\theta ,\lambda )}{k\ln (\lambda /\kappa )}}} \sum _{\ell =1}^{\infty } { e^{-\frac{p(\sigma ,\theta ,\lambda )+\ell }{k\ln (\lambda /\kappa )}}} \lesssim \lambda ^{-\beta } \ln (\lambda )^2 k^2 e^{-\frac{p(\sigma ,\theta ,\lambda )}{k\ln (\lambda /\kappa )}}, \end{aligned}$$

where we used the elementary estimate $1-e^{-2x} > rsim \min (x,1)$ for $x \ge 0$.

Applying Proposition 3.2 and inserting this estimate for the pole-contributions gives:

$$\begin{aligned} \lambda ^{r} E^{\lambda }(z^{-\beta })&=E^{\lambda }(\lambda ^{r} z^{-\beta }) \\&{\mathop {\lesssim }\limits ^{{Prop.~3.2}}} \quad \frac{e^{-2\pi d_\lambda /k}}{1-e^{-2\pi d_\lambda /k}} N(\lambda ^rg_{\lambda }^{\beta },{\mathcal {D}}_{d_\lambda }) + \lambda ^{-\beta +r} \ln (\lambda )^2 k^2 e^{-\frac{p(\sigma ,\theta ,\lambda )}{k\ln (\lambda /\kappa )}}. \end{aligned}$$

The bound from Lemma 3.6(iii) then completes the proof. $\square$

The previous estimate gives (almost) exponential convergence with respect to $\mathcal {N}_{\text {q}}$. But the rate of the exponential deteriorates like $1/\ln (\lambda )$ for large $\lambda$. In the following corollary, we give a $\lambda$-robust version of this estimate. We allow for an additional factor $\lambda ^{\rho }$ which will allow us to make use of possible additional smoothness when considering function-valued integrals.

#### Corollary 3.8

Fix $\lambda _0> \kappa >0$, $\overline{\beta } \in (0,1]$ and $r \in [0,\overline{\beta }/2]$. Then, for every $\varepsilon \ge 0$, there exist constants $C>0$, $\gamma >0$ such that for $\lambda > \lambda _0$, $\beta >\overline{\beta }$, $\rho \ge 0$, $k>0$, $\mathcal {N}_{\text {q}}\in \mathbb {N}$, the following estimate holds

3.15 $$\begin{aligned} \lambda ^{r}\Big |E^{\lambda }(z^{-\beta },\mathcal {N}_{\text {q}})\Big |\lesssim & {} \exp \Big (-\frac{[p(\sigma ,\theta )-\varepsilon ] \sqrt{\beta +\rho -r}}{\sqrt{k}}\Big ) \lambda ^{\rho }+ \exp (-\frac{\gamma }{k}) \nonumber \\{} & {} + \exp (- \gamma e^{k \mathcal {N}_{\text {q}}}). \end{aligned}$$

where the rate is given by (2.6). For $\varepsilon >0$, the implied constant in the estimate and $\gamma$ may depend on $\lambda _0$, $\sigma$, $\theta$, $\overline{\beta }$, $\kappa$. If $\varepsilon =0$, the constants in addition depend on $\rho$ and $\beta$.

#### Proof

We first show the estimate for $\varepsilon >0$. We note that for $\ln (\lambda /\kappa ) \ge k^{-1}$, we can bound the error in Theorem 3.7 by $\exp (-\gamma /k)$ (for an appropriate choice of constant $\gamma$) due to the smallness of the term $\lambda ^{-\beta }$. Thus it remains to consider the case $\ln (\lambda /\kappa ) < k^{-1}$. Similarly, if $\ln (\lambda )\le \max (\frac{c_2}{\varepsilon },-\ln (\kappa )\frac{p(\sigma ,\theta )-2\varepsilon }{\varepsilon },1)=:\mu _0$, the leading error term behaves like $\exp (-\gamma \frac{\mu _0}{k})$. We are left to consider the remaining case. Writing $\mu :=\ln (\lambda )$, the error term can be estimated:

3.16 $$\begin{aligned}{} & {} k^2 \ln (\lambda /\kappa )^2\lambda ^{-\beta +r-\rho } e^{-\frac{p(\sigma ,\theta ,\lambda )}{\max (1,\ln (\lambda /\kappa )) k}} \, \lambda ^{\rho } \nonumber \\{} & {} \quad \lesssim \exp \Big (\!\!-(\beta +\rho -r) \mu - \frac{p^2(\sigma ,\theta )/4 - \frac{c_2}{\mu }}{(\mu -\ln (\kappa )) k}\Big ) \lambda ^\rho \nonumber \\{} & {} \quad \lesssim \exp \Big (\!\!-(\beta +\rho -r) \mu - \frac{p^2(\sigma ,\theta ) - 2\varepsilon }{4\mu k}\Big )\lambda ^\rho . \end{aligned}$$

We look for the minimum of the exponent. Setting the derivative of the map

$$\begin{aligned} \mu \mapsto -(\beta +\rho -r) \mu - \frac{p^2(\sigma ,\theta )- 2\varepsilon }{4\mu k} \end{aligned}$$

to zero, we get that the minimum satisfies

$$\begin{aligned} 0=-(\beta +\rho -r) \mu _{\min }^2 + \frac{p^2(\sigma ,\theta ) - 2\varepsilon }{4k}, \quad \text {or} \quad \mu _{\min }:=\sqrt{ \frac{1}{(\beta +\rho -r)} \frac{p^2(\sigma ,\theta ) - 2\varepsilon }{4k}}. \end{aligned}$$

Inserting this value into (3.16) gives the stated result (after slightly changing $\varepsilon$ to get to the stated form).

To see the case for $\varepsilon =0$, we note that if $\ln (\lambda /\kappa ) \le \frac{\gamma _1 k^{-1/2}}{p(\sigma ,\theta )\sqrt{\beta +\rho -r}}$, we can estimate for the leading term in Theorem 3.7:

$$\begin{aligned} k^2 \ln (\lambda /\kappa )^2 \lambda ^{-\beta +r-\rho } e^{-\frac{\gamma _1}{\mu k}} \, \lambda ^{\rho }&\le k^2 \ln (\lambda /\kappa )^2 \lambda ^{-\beta +r-\rho } e^{-\frac{p(\sigma ,\theta )\sqrt{\beta +\rho -r}}{\sqrt{k}}} \, \lambda ^{\rho }. \end{aligned}$$

In the remaining case, we can estimate the higher order term in the $\ln (\lambda /\kappa )$-asymptotics as

$$\begin{aligned} e^{\frac{c_2}{\ln (\lambda /\kappa )^2 k}} \le e^{\frac{c_2 p(\sigma ,\theta ) \sqrt{\beta +\rho -r}}{\gamma _1}} =: C(\sigma ,\theta ,\beta ,\rho ). \end{aligned}$$

We can also write

$$\begin{aligned} \lambda ^{-\beta +r-\rho }= \kappa ^{-\beta +r-\rho }\Big ( \frac{\lambda }{\kappa }\Big )^{-\beta +r-\rho } \end{aligned}$$

and continue as in the proof for $\delta >0$ but using $\mu :=\ln (\lambda /\kappa )$. This time we no longer have to compensate for the factors involving $c_2/\mu$ and $-\ln (\kappa )$ by slightly reducing the rate. The price we pay is that the constant may blow up for $\rho \rightarrow \infty$. $\square$

We can now leverage our knowledge about the function $g^\beta _\lambda$ to gain insight into the discretization error for (2.5). This allows us to prove the two main theorems of this section. First we deal with the finite regularity case.

#### Proof of Theorem 2.5

Let $(\lambda _j, v_j)_{j=0}^{\infty }$ denote the eigenvalues and eigenfunctions of the self-adjoint operator $\mathcal {L}$. Just as we did in the proof of Corollary 3.5, we plug the eigen-decomposition into the Riesz–Dunford calculus and Definition 2.1 to get for the discretization error:

$$\begin{aligned} \left\| u-u_{k}\right\| _{{\mathbb {H}}^{2r}}^2&=\sum _{j=0}^{\infty }{ \Big |(1+\lambda _j^{r})\frac{1}{2\pi i}\int _{\mathcal {C}}{g^\beta _{\lambda _j}(y) \,dy} - \frac{1}{2\pi i}\sum _{n=-\mathcal {N}_{\text {q}}}^{\mathcal {N}_{\text {q}}}{g^\beta _{\lambda _j}(k\,n)}\Big |^2 \left| \big (f,v_j\big )_{{\mathcal {X}}}\right| ^2}. \end{aligned}$$

Applying Corollary 3.8 then gives for $\rho \ge 0$

$$\begin{aligned} \left\| u-u_{k}\right\| _{{\mathbb {H}}^{2r}}^2&\lesssim e^{-\frac{2[p(\sigma ,\theta )-\varepsilon ] \sqrt{\beta +\rho -r}}{\sqrt{k}}} \sum _{j=0}^\infty {\lambda _j^{2\rho } \left| \big (f,v_j\big )_{{\mathcal {X}}}\right| ^2} +\big [e^{-\frac{\gamma }{k}} + e^{- \gamma e^{k \mathcal {N}_{\text {q}}}} \big ]^2\left\| f\right\| ^2_{{\mathcal {X}}} \\&\lesssim e^{-\frac{2[p(\sigma ,\theta )-\varepsilon ] \sqrt{\beta +\rho -r}}{\sqrt{k}}} \left\| f\right\| ^2_{{\mathbb {H}}^{2\rho }} +\Big [e^{-\frac{\gamma }{k}} + e^{- \gamma e^{k \mathcal {N}_{\text {q}}}} \Big ]^2 \left\| f\right\| ^2_{{\mathcal {X}}}. \end{aligned}$$

$$\square $$

Next we prove the improved estimates for the case of $\mathcal {G}^{\mathcal {L}}(C_{f},R_{f},\omega )$-regularity.

#### Proof of Theorem 2.8

For simplicity of notation, we ignore the cutoff error, i.e., for now consider $\mathcal {N}_{\text {q}}=\infty$. The cutoff error can either be easily tracked throughout the proof or added at the end, analogously to Corollary 3.5.

We first note, that by Stirling’s formula, we can estimate the derivatives of *f* by

$$\begin{aligned} \left\| f\right\| _{{\mathbb {H}}^{\rho }}&\le \widetilde{C}_{f} \exp \big ( \rho (\omega \ln (\rho )+ c_2)\big ). \end{aligned}$$

By assumption, we can apply Theorem 2.5 for any $\rho \ge 0$. Picking $\rho =\frac{\delta }{k\ln (k)^2}$ for $\delta$ sufficiently small and $\varepsilon :=p(\sigma ,\theta )/2$ (because we need $\rho$-robust error estimates) gives:

3.17 $$\begin{aligned} \left\| u-u_{k}\right\| _{{\mathbb {H}}^{\beta }}&\lesssim \exp \Big (-\frac{p(\sigma ,\theta ) \sqrt{\beta /2+\delta k^{-1}{\left| \ln (k)\right| }^{-2}}}{2\sqrt{k}}\Big ) \left\| f\right\| _{{\mathbb {H}}^{\frac{2\delta }{k\ln (k)^2}}} +e^{-\frac{\gamma }{k}} \left\| f\right\| _{{\mathcal {X}}} \nonumber \\&\lesssim e^{-\!\frac{\sqrt{\delta } \gamma '}{k\left| \ln (k)\right| }} C_{f} e^{\frac{2\delta }{k\left| \ln (k)\right| ^2}\big (\omega \ln (\frac{2\delta }{k\left| \ln (k)\right| ^2})+ c_2\big )\!\!} +e^{-\frac{\gamma }{k}} \left\| f\right\| _{{\mathcal {X}}} \nonumber \\&\lesssim e^{-\frac{\sqrt{\delta } }{k\left| \ln (k)\right| }\big ( \gamma '- \frac{2\sqrt{\delta }}{\left| \ln (k)\right| }(\omega \ln \big (\frac{2\delta }{k\left| \ln (k)\right| ^2}\big )+ c_2\big )} +e^{-\frac{\gamma }{k}} \left\| f\right\| _{{\mathcal {X}}}. \end{aligned}$$

We need to show that the bracket in the exponential is positive. In order to do this, we expand the logarithmic term as

$$\begin{aligned} \ln \Big (\frac{2\delta }{k\left| \ln (k)\right| ^2}\Big )&= \ln (2\delta ) - \ln (k) - 2\ln (|\ln (k)|). \end{aligned}$$

This first term is negative, and for the others we note that

$$\begin{aligned} \frac{2\omega \sqrt{\delta }}{\left| \ln (k)\right| } \Big ( - \ln (k) - 2\ln (|\ln (k)|) + c_2\Big ) \end{aligned}$$

is uniformly bounded as $|\ln (|\ln (k)|)|$ grows slower than $|\ln (k)|$ as $k\rightarrow 0$. Due to the leading $\sqrt{\delta }$ term, we can make $\delta$ small enough (independently of *k*) to ensure that the second term in the exponent of (3.17) is smaller than $\gamma '$ and the statement follows. If $\omega =0$, we don’t have to compensate the factor $e^{\omega \rho \ln (\rho )}$, therefore picking $\rho \sim k^{-1}$ is sufficient and the improved statement follows. $\square$

### The parabolic problem

Now that the stationary problem is well understood, we can move on to analyzing the discretization of the time dependent problem introduced in Sect. 2.2.

#### The Mittag Leffler function

The representation (2.8) hints that it is crucial to understand the Mittag–Leffler function if one wants to analyze the time dependent problem (2.7). We follow [20, Section 1.8]. For parameters $\alpha >0$, $\mu \in \mathbb {R}$, the Mittag–Leffler function is an analytic function on $\mathbb {C}$ and given by the power series

3.18 $$\begin{aligned} e_{\alpha ,\mu }(z):=\sum _{n=0}^{\infty }{\frac{z^n}{\Gamma (n\alpha +\mu )}}. \end{aligned}$$

We collect some important properties we will need later on. We start with the following decomposition result, also giving us asymptotic estimates.

##### Proposition 3.9

For $0< \alpha < 2, \mu \in \mathbb {R}$ and $\frac{\alpha \pi }{2}< \zeta < \alpha \pi$, we can decompose the Mittag–Leffler function as

3.19 $$\begin{aligned} e_{\alpha ,\mu }(z)&= - \sum _{n=1}^{N}{\frac{1}{\Gamma (\mu -\alpha n)}\frac{1}{z^n}} + R_{\alpha ,\mu }^{N}(z) \qquad \text {for } \zeta \le \left| {\text {Arg}}{z}\right| \le \pi . \end{aligned}$$

where $R_{\alpha ,\mu }^{N}$ is analytic away from zero and satisfies

3.20 $$\begin{aligned} \left| R_{\alpha ,\mu }^{N}(z)\right| \le C\, \Gamma (\alpha N) \left| z\right| ^{-(N+1)} \qquad \forall \left| z\right| \ge z_0 > 0 \end{aligned}$$

for a constant $C>0$ depending only on $z_0$ and $\zeta$.

##### Proof

The statement can be found in [20, Eqn 1.8.28] where the dependence of the remainder term on *N* is not made explicit. To get the explicit estimate on the remainder, we follow [14, Section 18.1]. There, it is proven that the remainder can be written as

$$\begin{aligned} R_{\alpha ,\mu }^{N}(z)&=\frac{ z^{-N-1}}{2\pi i} \int _{\widetilde{\mathcal {C}}}{\Big (1-\frac{t^{\alpha }}{z}\Big )^{-1} t^{(N+1)\alpha -\mu } e^{t} \; dt}, \end{aligned}$$

where $\widetilde{\mathcal {C}}$ can be taken as two rays $\{r \zeta _0: \, r\ge 1\}$, $\{r \overline{\zeta _0}: \, r\ge 1\}$ and a small circular arc connecting the two without crossing the negative real axis. $\zeta _0$ is taken in the left half-plane such that the opening angle of $\widetilde{\mathcal {C}}$ is sufficiently large in order to avoid possible poles of the integrand and ensure that the term $(1-t^{\alpha }/z)^{-1}$ is uniformly bounded. The stated result then follows easily by comparing the integral under consideration to the definition of the Gamma function. $\square$

Setting $N=1$ in Proposition 3.9 and simple calculation yields the following estimates:

3.21 $$\begin{aligned} \left| e_{\alpha ,\mu }(z)\right| \le \frac{C}{1+\left| z\right| ^{s}} \quad \text {for } \zeta \le \left| {\text {Arg}}{z}\right| \le \pi ,\, s \in [0,1] \end{aligned}$$

For $\alpha =\mu =1$, the Mittag–Leffler function $e_{1,1}$ is the usual exponential function. For the decomposition result, we can skip the terms involving powers $z^{-n}$ in this case as $e^{z}$ already decays faster than any polynomial.

Finally, we need a way of computing antiderivatives of the convolution kernel in (2.8).

##### Proposition 3.10

For $n \in \mathbb {N}_0$, $\alpha >0$, $z \in \mathbb {C}\setminus \{0\}$, $\lambda \in \mathbb {C}$, it holds that

3.22 $$\begin{aligned} z^{\alpha -1}e_{\alpha ,\alpha }(\lambda \, z^{\alpha } )&= \Big (\frac{\partial }{\partial z}\Big )^{n} \Big (z^{\alpha +n-1} e_{\alpha ,\alpha +n}(\lambda \,z^{\alpha }) \Big ). \end{aligned}$$

##### Proof

Follows from [20, Eqn. 1.10.7] by taking $\beta :=\alpha +n$. $\square$

#### Double exponential quadrature for the parabolic problem

***The case of finite regularity*** In this section, we investigate the convergence of our method in the case that $u_0$ and *f* have finite ${\mathbb {H}}^{2\rho }$-regularity for some $\rho \ge 0$. It will showcase most of the new ingredients needed to go from the elliptic case to the time dependent one while keeping the technicalities to a minimum. The step towards Gevrey-regularity will then mainly consist of carefully retracing the argument and fine-tuning parameters. We start with the case if $f=0$.

##### Lemma 3.11

Assume that either $\alpha +\beta <2$ or $\sigma =1$ (i.e., the case $\alpha =\beta =1$ and $\sigma =1/2$ is not allowed). Let $u(t):=e_{\alpha ,\mu }(-t^{\alpha } \mathcal {L}^{\beta }) u_0$ and assume $u_0 \in {\mathbb {H}}^{2\rho }(\Omega )$ for some $\rho >0$. Let $u_k:=Q^{\mathcal {L}}\big (e_{\alpha ,\mu }(-t^{\alpha } z^{\beta }),\mathcal {N}_{\text {q}}\big ) u_0$ be the corresponding discretization using stepsize $k>0$ and $\mathcal {N}_{\text {q}}\in \mathbb {N}$ quadrature points.

Then, the following estimate holds for all $\eta \ge 1$ and $r\in [0,\beta /2]$:

$$\begin{aligned} \left\| u(t)-u_{k}(t)\right\| _{{\mathbb {H}}^{2r}}\lesssim & {} t^{-\eta \alpha } \Big ( e^{ -\min {\big \{p(\sigma ,\theta )\sqrt{\beta +\rho -r} ,\sqrt{\beta \eta } \,\gamma _1\big \}} \frac{1}{\sqrt{k}}} + e^{-\frac{\gamma }{k}}\Big ) \left\| u_0\right\| _{{\mathbb {H}}^{2\rho }} \\{} & {} + t^{-\alpha /2} \exp (-\gamma e^{k \mathcal {N}_{\text {q}}}) \left\| u_0\right\| _{{\mathbb {H}}^{2\rho }}. \end{aligned}$$

Here $\gamma _1$ is the constant from Corollary 3.5. The implied constant and $\gamma$ may depend on *r*, the smallest eigenvalue $\lambda _0$ of $\mathcal {L}$, $\beta$,$\alpha$, $\kappa$, $\theta$, $\sigma$ and $\rho$.

##### Proof

We start with $\mathcal {N}_{\text {q}}=\infty$ and split the Mittag–Leffler function according to (3.19). We write

3.23 $$\begin{aligned} E^{\mathcal {L}}\Big (e_{\alpha ,\mu }(-t^{\alpha } z^{\beta }) \Big )&= \sum _{n=1}^{N}{\frac{ (-1)^n \,t^{-\alpha n} }{\Gamma (\mu -\alpha n)} E^{\mathcal {L}}(z^{-\beta n})} + E^{\mathcal {L}}\big (R^N_{\alpha ,\mu }(-t^{\alpha } z^{\beta })\big ). \end{aligned}$$

For the first terms, we apply Theorem 2.5, and for the final term we use the decay estimate (3.20) and Corollary 3.5. Note that this is where we have to exclude the case $\alpha =\beta =1$ and $\sigma =1/2$. If $\alpha <1$ the Mittag–Leffler function is contractive on a large enough sector. If $\beta <1$, the map $z\mapsto z^{\beta }$ maps the required sector into the right half plane. Otherwise, the exponential function only decays in the right half-plane, not any slightly bigger sector. Thus, if $\sigma =1/2$, Corollary 3.5 does not apply.

Overall, we get the estimate:

$$\begin{aligned} \left\| E^{\mathcal {L}}\Big (e_{\alpha ,\mu }(t^{\alpha } z^{\beta }) \Big )\right\| _{{\mathbb {H}}^{2 r}}&\lesssim \sum _{n=1}^{N-1}{\frac{ t^{-\alpha n} }{\Gamma (\mu -\alpha n)} e^{- \min \big \{\frac{p(\sigma ,\theta ) \sqrt{\beta n + \rho -r}}{\sqrt{k}}, \frac{\gamma }{k}\big \} }\left\| u_0\right\| _{{\mathbb {H}}^{2\rho }}} \\&\quad \ + \Gamma (\alpha N) t^{-\alpha N} e^{-\gamma _1 \frac{\sqrt{\beta (N+1) + \rho -r -2\varepsilon }}{\sqrt{k}}} \left\| u_0\right\| _{{\mathbb {H}}^{2\rho }}. \end{aligned}$$

To simplify the calculations, we make use of the fact that $\beta - r - 2\varepsilon \ge \beta /2 - 2\varepsilon >0$ and $\rho >0$. That way, the last term can be simplified to

$$\begin{aligned} \Gamma (\alpha N) t^{-\alpha N} \exp \Big (-\gamma _1 \frac{\sqrt{\beta N}}{\sqrt{k}}\Big ) \left\| u_0\right\| _{{\mathbb {H}}^{2\rho }}. \end{aligned}$$

If $\eta$ is an integer, we can pick $N=\eta$ to get the statement for $\mathcal {N}_{\text {q}}=\infty$. For general $\eta \ge 1$, we can interpolate between $\lfloor \eta \rfloor$ and $\lfloor \eta \rfloor +1$. The treatment of the cutoff error follows as in Corollary 3.5, exploiting that $e_{\alpha ,\mu }(z)$ decays like (3.21) with $s:=\beta /2$. $\square$

Picking $\eta$ large enough, Lemma 3.11 shows that for fixed times $t>0$ we get the same convergence rate as for the elliptic problem, though the approximation deteriorates as *t* gets small.

Now that we understand the homogeneous problem, we can look at the case of allowing inhomogeneous right-hand sides *f* by using the representation formula (2.8), and finally prove the main result Theorem  2.10. We point out that naive application of Corollary (3.16) also inside the time-convolution integral would fail to give good rates, as the error may blow up faster than $\tau ^{-\alpha }$ for small times, leading to a non-integrable function. Instead, the following proof relies on integration by parts and (3.22) to split the convolution into point evaluations similar to Lemma 3.11 and an integrable remainder term.

##### Proof of Theorem 2.10

As we have already estimated the error of the homogeneous part, we only consider the part corresponding to the inhomogenity, i.e., for now let $u_0=0$. We integrate by parts *m* times, using (3.22):

$$\begin{aligned} \int _{0}^{t} { \tau ^{\alpha -1} e_{\alpha ,\alpha }\big (-\tau ^{\alpha } \lambda ^\beta \big ) f(t-\tau )\,d\tau }= & {} {\sum _{j=1}^{m} { t^{\alpha +j-1} e_{\alpha ,\alpha +j}(- t^{\alpha } \lambda ^{\beta } ) f^{(j-1)}(0)}}\\{} & {} +{\int _{0}^{t} { \tau ^{\alpha +m-1} e_{\alpha ,\alpha +m}\big (-\tau ^{\alpha } \lambda ^\beta \big ) f^{(m)}(t-\tau )\,d\tau }} \end{aligned}$$

Transferring this identity to the operator-valued setting, this means that we can analyze the quadrature error for these terms separately.

3.24 $$\begin{aligned} \left\| u(t)-u_k(t)\right\| _{{\mathbb {H}}^{2r}}&=\big \Vert \int _{0}^{t} { \tau ^{\alpha -1}E^\mathcal {L}\Big (e_{\alpha ,\alpha }\big (-\tau ^{\alpha } z^\beta \big )\Big ) f(t-\tau )\,d\tau } \big \Vert _{{\mathbb {H}}^{2r}} \nonumber \\&\le {\sum _{j=1}^{m} { t^{\alpha +j-1} \big \Vert { E^{\mathcal {L}}\big (e_{\alpha ,\alpha +j}(- t^{\alpha }z^{\beta } ) \big )f^{(j-1)}(0)}\big \Vert _{{\mathbb {H}}^{2r}}}} \nonumber \\&\quad + {\int _{0}^{t} { \tau ^{\alpha +m-1} \big \Vert {E^{\mathcal {L}}\big (e_{\alpha ,\alpha +m}\big (-\tau ^{\alpha } z^\beta \big )\big ) f^{(m)}(t-\tau )}\big \Vert _{{\mathbb {H}}^{2r}}\,d\tau }}. \end{aligned}$$

All the terms appearing are of the structure in Lemma 3.11. Most notably, the first *m* terms are evaluated at a fixed $t>0$ thus we don’t have to analyze them further and can just accept some *t*-dependence.

Investigating the remaining integral, we get by using $\eta :=m/\alpha +q$ in Lemma 3.11:

$$\begin{aligned}{} & {} \int _{0}^{t} { \tau ^{\alpha +m-1} \left\| E^{\mathcal {L}}\big (e_{\alpha ,\alpha +m}(-\tau ^{\alpha } z^\beta )\big ) f^{(m)}(t-\tau )\right\| _{{\mathbb {H}}^{2r}}\,d\tau } \\{} & {} \quad \lesssim \int _{0}^{t}{\tau ^{\alpha +m-1-m-\alpha q} \Big [\!e^{-\frac{ \min \{p(\sigma ,\theta ) \sqrt{\beta +\rho -r},\gamma _1\sqrt{\eta }\}}{\sqrt{k}}} \!+\!e^{-\frac{\gamma }{k}} \Big ]\big \Vert {f^{(m)}(t-\tau )}\big \Vert _{{\mathbb {H}}^{2\rho }} d\tau }. \end{aligned}$$

For $q<1$, this is an integrable function (with respect to $\tau$) and the integral grows like $t^{\alpha (1-q)}$.

We now focus on extracting the correct *t* dependencies. For small times, the dominating *t*-dependence in the estimates above can be found in the first term of (3.24), which behaves like $t^{-m\alpha (1-q)}$. If we put back the homogeneous contribution from Lemma 3.11, this term will dominate for small times like $t^{-m- q\alpha }$. For larger times, the initial error term in (3.24) is dominant, giving behavior $T^{\alpha }$. The cutoff error is treated like before, making use of the decay of $e_{\alpha ,\alpha }$. We just point out that the homogeneous cutoff error behaves like $t^{-\alpha /2}$ and the inhomogeneous part $t^{\alpha /2}$. We crudely estimated both by $\max (t^{-m-q\alpha },T^{\alpha })$ to simplify the statement of the theorem). $\square$

##### Remark 3.12

Corollary 2.10 shows that, as long as we assume that *f* is smooth enough in time we recover the same convergence rate $p(\sigma ,\theta )\sqrt{\beta +\rho -r}$ as in the homogeneous and elliptic case. $\square$

***The case of Gevrey-type regularity*** If the data not only satisfies some finite regularity estimates but instead is even in some Gevrey-type class of functions, we can again improve the convergence rate, and almost get rid of the square root in the exponent. We go back to the homogeneous problem and assume that $k<1/2$ so that the logarithmic terms can be written down succinctly.

##### Lemma 3.13

Assume that either $\alpha +\beta <2$ or $\sigma =1$ (i.e., the case $\alpha =\beta =1$ and $\sigma =1/2$ is not allowed). Let $u(t):=e_{\alpha ,\mu }(-t^{\alpha } \mathcal {L}^{\beta }) u_0$ and assume that there exist constants $C_{u_0} , \omega , R_{u_0}>0$ such that

$$\begin{aligned} \left\| u_0\right\| _{{\mathbb {H}}^{\rho }}&\le C_{u_0} \, R_{u_0}^{\rho } \,\big (\Gamma (\rho +1)\big )^{\omega } < \infty \qquad \forall \rho \ge 0. \end{aligned}$$

Let $u_k(t):=Q^{\mathcal {L}}\big (e_{\alpha ,\mu }(-t^{\alpha } z^{\beta }),\mathcal {N}_{\text {q}}\big ) u_0$ be the discretization of *u* using stepsize $k\in (0,1/2)$ and $\mathcal {N}_{\text {q}}\in \mathbb {N}$ quadrature points. Then, the following estimate holds:

$$\begin{aligned} \left\| u(t)-u_{k}(t)\right\| _{{\mathbb {H}}^{\beta }}&\lesssim C_{u_0} \exp \big (-\frac{\gamma }{k\left| \ln (t_\star )\right| \left| \ln {k}\right| }\big ) + C_{u_0}t^{-\alpha /2}\exp \big (- \gamma e^{k \mathcal {N}_{\text {q}}}\big ). \end{aligned}$$

with $t_{\star }:=\min (t,1/2)$. The implied constant and $\gamma$ may depend on $\varepsilon$, the smallest eigenvalue $\lambda _0$ of $\mathcal {L}$, $\beta$, $\alpha$, $\kappa$, $\theta$, $\sigma$, $R_{u_0}$ and $\omega$.

##### Proof

We go back to (3.23), but apply Theorem 2.8 to each of the first *N* terms, getting:

3.25 $$\begin{aligned} \begin{aligned} \left\| E^{\mathcal {L}}\Big (e_{\alpha ,\mu }(-t^{\alpha } z^{\beta }) \Big ) u_0\right\| _{{\mathbb {H}}^{\beta }}&\lesssim C_{u_0}\sum _{n=1}^{N}{\frac{ t^{-\alpha n} }{\Gamma (\mu -\alpha n)} \exp \Big (-\frac{\gamma }{k \left| \ln (k)\right| }\Big ) } \\&\quad \ + \Gamma (\alpha N) t^{-\alpha N} \exp \Big (-\gamma \frac{\sqrt{N-2\varepsilon }}{\sqrt{k}}\Big ) \left\| u_0\right\| _{{\mathcal {X}}}. \end{aligned} \end{aligned}$$

We estimate the first *N* terms by

$$\begin{aligned} \frac{ t^{-\alpha n} }{\Gamma (\mu -\alpha n)} \exp \Big (-\frac{\gamma }{k \left| \ln (k)\right| }\Big )&\lesssim \exp \Bigg (-\alpha \ln (t) n + c_1 n \log (n) -\frac{\gamma }{k \left| \ln (k)\right| }\Bigg ). \end{aligned}$$

For $n \le \frac{\delta }{k\left| \ln (t_{\star })\right| ^2\left| \ln (k)\right| ^2}$, we can estimate the exponent by

$$\begin{aligned} -\frac{1}{k \left| \ln (t_\star )\right| \left| \ln (k)\right| } \Bigg [ {-\frac{\alpha \delta }{\left| \ln (k)\right| } } - \frac{ c_1 \delta }{\left| \ln (t_\star )\right| \left| \ln (k)\right| } \ln \Big (\frac{\delta }{\ln (t_\star )^2 \ln (k)^2 k}\Big ) +\gamma \ln (2) \Bigg ] \end{aligned}$$

For $\delta$ small enough, depending on $c_1$, $\alpha$ and $\gamma$, the term in brackets is uniformly positive (i.e., independently of *t* and *k*), we can thus estimate for some $\gamma _1>0$:

$$\begin{aligned} \frac{ t^{-\alpha n} }{\Gamma (\mu -\alpha n)} \exp \Big (-\frac{\gamma }{k \left| \ln (k)\right| }\Big )&\lesssim e^{-\frac{\gamma _1}{k\left| \ln (t_\star )\right| \left| \ln (k)\right| }}. \end{aligned}$$

The remainder term behaves like

$$\begin{aligned} t^{-\alpha N} \Gamma (\alpha N) \exp \Big (-\gamma \frac{\sqrt{N-2\varepsilon }}{\sqrt{k}}\Big )&\lesssim \exp \Big (\!\!-\alpha N \ln (t) - c_2 N \ln (N) - \gamma \frac{\sqrt{N-2\varepsilon }}{\sqrt{k}}\Big ). \end{aligned}$$

By picking $N=\big \lceil {\frac{\delta }{k\left| \ln (t_\star )\right| ^2\left| \ln (k)\right| ^2}}\big \rceil$, the exponent be bounded up to a constant by

$$\begin{aligned} -\frac{\sqrt{\delta }}{k \left| \ln (t_\star )\right| \left| \ln (k)\right| } \Bigg [ {-\frac{\gamma \sqrt{\delta }}{\left| \ln (k)\right| } - \frac{c_1\sqrt{\delta }}{\left| \ln (t_\star )\ln (k)\right| } \ln \Big (\frac{\delta }{k \left| \ln (t_\star )\right| ^2 \left| \ln (k)\right| ^2}\Big ) + \gamma \ln (2) } \Bigg ]. \end{aligned}$$

By taking the factor $\delta$ sufficiently small, we get that the term in brackets stays uniformly positive, which shows

$$\begin{aligned} \left\| E^{\mathcal {L}}\Big (e_{\alpha ,\mu }(-t^{\alpha } z^{\beta }) \Big )\right\| _{{\mathbb {H}}^{\beta }}&\lesssim \exp \Big ({-\frac{\gamma }{\left| \ln (t_\star )\right| \left| \ln (k)\right| k}}\Big ). \end{aligned}$$

The cutoff error can easily be dealt with as in the previous results, as the Mittag–Leffler function satisfies the decay bound (3.21) for $s=1/2$. $\square$

Finally, we are in a position to also include the inhomogenity *f* into our treatment. This means we can prove the main result Theorem 2.11. Just as in Lemma 2.10, we use integration by parts to decompose the error into parts for positive times and a remainder integral with “nice enough” behavior with respect to $\tau$.

##### Proof of Theorem 2.11

We again work under the assumption $u_0=0$ and focus on the error when dealing with the inhomogenity *f* alone and also start with $\mathcal {N}_{\text {q}}=\infty$. We also for now take $t\le 1$.

Going back to (3.24) we get for $N \in \mathbb {N}_0$ to be fixed later

3.26 $$\begin{aligned} \begin{aligned} \left\| u(t)-u_k(t)\right\| _{{\mathbb {H}}^{2r}}&\le {\sum _{j=1}^{N} { t^{\alpha +j-1} \big \Vert { E^{\mathcal {L}}\big (e_{\alpha ,\alpha +N}(-t^{\alpha } z^{\beta }) \big )f^{(j-1)}(0)}\big \Vert _{{\mathbb {H}}^{\beta }}}}\\&\quad +{\int _{0}^{t} { \tau ^{\alpha +N-1} \big \Vert {E^{\mathcal {L}}\big (e_{\alpha ,\alpha +n}(-\tau ^{\alpha } z^\beta )\big ) f^{(N)}(t-\tau )}\big \Vert _{{\mathbb {H}}^{\beta }}\,d\tau }}. \end{aligned} \end{aligned}$$

For the first terms, we apply Lemma 3.13 to get exponential convergence, as long as $f^{(j)}$ is in the right Gevrey-type class. Namely, we note that we can estimate

$$\begin{aligned} \left\| {f}^{(n)}(t)\right\| _{{\mathbb {H}}^{2\rho }} \lesssim e^{\widetilde{\omega } N \ln (N)} e^{\widetilde{\omega } \rho \ln (\rho )} \end{aligned}$$

by possibly tweaking $\widetilde{\omega }$ compared to $\omega$. This allows us to estimate

3.27 $$\begin{aligned} {\sum _{j=1}^{N} { t^{\alpha +j-1} \left\| E^{\mathcal {L}}\Big (e_{\alpha ,\alpha +j}(- t^{\alpha } z^{\beta } ) \Big )f^{(j-1)}(0)\right\| _{{\mathbb {H}}^{2r}}}}&\lesssim e^{\widetilde{\omega }N \ln (N)} e^{-\frac{\gamma }{\left| \ln (t_\star )\right| \left| \ln (k)\right| k}}. \end{aligned}$$

Again restricting $\widetilde{\omega }$ to absorb the factor *N* due to the summation.

For the remainder in (3.26), we look at the pointwise error at fixed $0<\tau <t$, shortening $\widetilde{f}^{(N)}:=f^{(N)}(t-\tau )$. Going back to (3.25), we can use the additional powers of *t* to get rid of the $\ln (t)$ term in the exponential:

$$\begin{aligned}{} & {} \tau ^{\alpha +N-1}\left\| E^{\mathcal {L}}\big (e_{\alpha ,\alpha +n}\big (-\tau ^{\alpha } z^\beta \big )\big ) \widetilde{f}^{(N)}\right\| _{{\mathbb {H}}^{\beta }} \\{} & {} \quad \lesssim \sum _{n=1}^{N-1}{C_{f} e^{\widetilde{\omega } N \ln (N)}\frac{ \tau ^{-\alpha (n-1) + N -1} }{\Gamma (\mu -\alpha n)} \exp \Big (-\frac{\gamma }{k \left| \ln (k)\right| }\Big ) } \\{} & {} \qquad + \Gamma (\alpha N) \tau ^{(1-\alpha )(N-1)} C_f e^{\widetilde{\omega } N \ln (N)} \exp \Big (-\gamma \frac{\sqrt{N-2\varepsilon }}{\sqrt{k}}\Big ). \end{aligned}$$

We then proceed as in the proof of Lemma 3.13, noting that since the $\tau$-dependent terms can be bounded independently of *N* we can get by without the $\ln (t_\star )$-term in the exponent. Overall, we get by tuning $N \sim \delta /(\left| \ln (k)\right| ^2 k)$ (also in (3.27)) appropriately:

$$\begin{aligned} \left\| u(t)-u_k(t)\right\| _{{\mathbb {H}}^{2r}}&\lesssim \exp \Big (-\frac{\gamma }{k \left| \ln (t_\star )\right| \left| \ln (k)\right| }\Big ) + \int _{0}^{t}{\exp \Big (-\frac{\gamma }{k \left| \ln (k)\right| }\Big ) \,d\tau }. \end{aligned}$$

which easily gives the stated result. If $t>1$, we can skip the integration by parts step for the integration over (1, *t*) and directly apply Lemma 3.13. The cutoff error is treated as always. $\square$

## Numerical examples

In this section, we investigate, whether the theoretical results obtained in Sects. 3.2 and 3.3 can also be observed in practice. We compare the following quadrature schemes:

(i) DE1: double exponential quadrature using $\sigma =1/2$ and $\theta =4$,
(ii) DE2: double exponential quadrature using $\sigma =1$ and $\theta =4$,
(iii) DE3: double exponential quadrature using $\sigma =1$ and $\theta =1$,
(iv) sinc: standard sinc quadrature
(v) Balakrishnan: a quadrature scheme based on the Balakrishnan formula
(vi) BURA: best uniform rational approximation

For the double exponential quadrature schemes, we used $k=0.9\ln (r\mathcal {N}_{\text {q}})/\mathcal {N}_{\text {q}}$ with $r:=1$ for $\beta >0.4$ and $r:=5$ for $\beta <0.4$. This makes the cutoff error decay like $e^{- \beta r \mathcal {N}_{\text {q}}^{0.9}}$, which is sufficiently fast to not impact the overall convergence rate. The factor 0.9 was observed to have some slightly improved stability compared to 1. The damping constant *r* was introduced to get good behavior for small $\beta$; see Sect. 4.3.

For the standard sinc-quadrature, the proper tuning of *k* and $\mathcal {N}_{\text {q}}$ is more involved. Following [4], we picked $k=\sqrt{\frac{2\pi d}{\beta \mathcal {N}_{\text {q}}}}$ with $d=\pi /5$. The Balakrishnan formula is only possible for the elliptic problem. It is described in detail in [5]. Following [5, Remark 3.1] we used

$$\begin{aligned} k:=\sqrt{\frac{\pi ^2}{1.8\beta N}} \qquad M:=\left\lceil \frac{\pi ^2}{2(1-\beta )k^2}\right\rceil , \end{aligned}$$

where *M* is the number of negative quadrature points. This corresponds (in their notation) to taking $s^+:=\beta /10$, which was taken because it yielded good results (Fig. 4).

### The pure quadrature problem

In this section, we focus on a scalar quadrature problem. Namely, we investigate how well our quadrature scheme can approximately evaluate the following functions using the Riesz–Dunford calculus (a) $z^{-\beta }$ and (b) $e_{\alpha ,1}(-t^{\alpha } z^{\beta })$ at different values $\lambda \in (4,\infty )$. This is equivalent to solving the elliptic and parabolic problem with data consisting of a single eigenfunction corresponding to the eigenvalue $\lambda$. Throughout, we used $\kappa :=3$. Theoretical investigations revealed, that the quadrature error is largest at $\ln (\lambda ) \sim k^{-1/2}$ (see the proof of Corollary 3.8). Therefore, we make sure that for each *k* under consideration, such a value of $e^{\frac{1}{\sqrt{k}}}$ is among the $\lambda$-values sampled. More precisely, the sample points consist of

$$\begin{aligned} \bigcup _{\mathcal {N}_{\text {q}}=1}^{N_{\max }}\big \{ 5+\exp \big (2\sqrt{\beta /k(\mathcal {N}_{\text {q}})}\big ) \big \} \cup \big \{5+\exp (\beta /k(\mathcal {N}_{\text {q}})) \big \}, \end{aligned}$$

with $k(\mathcal {N}_{\text {q}})=0.9 \ln (\mathcal {N}_{\text {q}})/\mathcal {N}_{\text {q}}$, and we consider the maximum error over all these samples. We used $t:=1$ for all experiments.

**Fig. 1:**
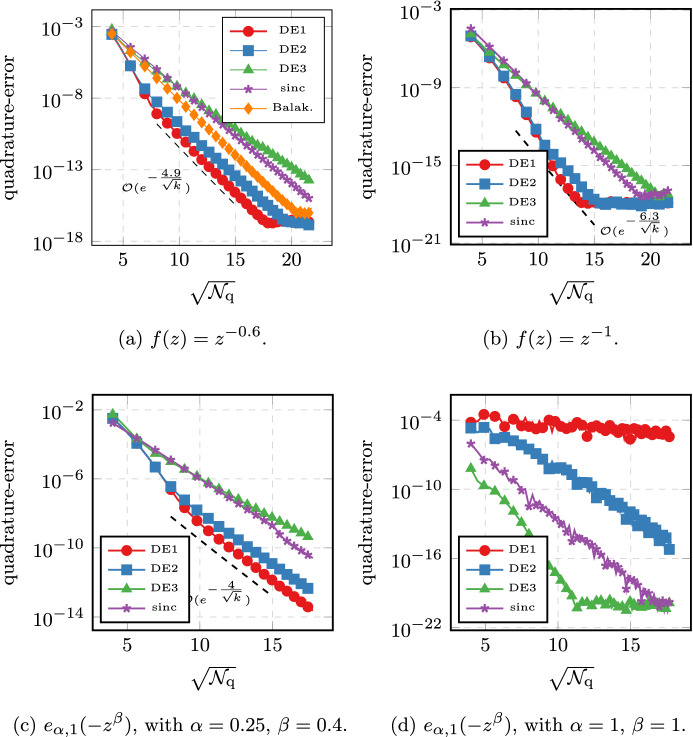
Comparison of quadrature schemes—scalar problem

We observe that for the most part, choosing $\sigma =1/2$ and $\theta$ moderately large gives the best result. This agrees with our theoretical findings. This method fails to converge though if $\alpha =\beta =1$ is chosen as the parameters for the Mittag–Leffler function. This also agrees with the theory, because in this case, $\psi _{\sigma ,\theta }$ fails to map into the domain where $e_{\alpha ,\mu }$ is decaying (see (3.21)). This shows that the restriction on $\sigma$ in the theorems of Sect. 3.3 is necessary. If we only consider the elliptic problem, no such restriction is necessary, as the decay property is valid on all of the complex plane. All the other methods perform well in all of the cases. The straight-forward double exponential formula, i.e., $\sigma =\theta =1$, is often outperformed by the simple sinc quadrature scheme, (except in the $\alpha =\beta =1$ case of the exponential). For comparison, we’ve included the (rounded) predicted rate for the DE1 scheme in the plots. We observe that for several applications our estimates appear sharp. For $f(z)=z^{-1}$ the scheme outperforms the prediction, but this might be due to a large preasymptotic regime. We note that for $e^{-z^{\beta }}$, we expect better estimates than the ones presented in this article to be possible due to the exponential decay. This is also true for the standard sinc methods, see [3].

**Fig. 2:**
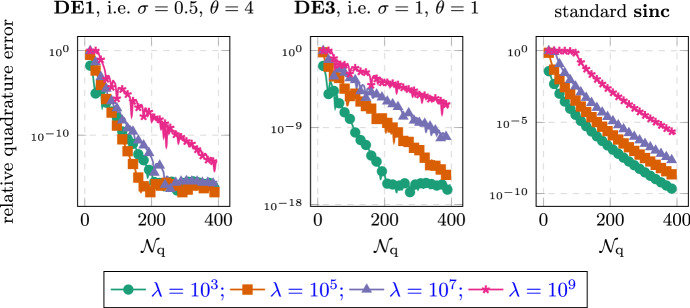
Comparison of $\lambda$ dependence for different quadrature schemes

Second, we look at the case of a single frequency $\lambda$ and see how the convergence rate decays as $\lambda \rightarrow \infty$. In order to better see the $\lambda$-dependence of the quadrature error, we consider the relative error of the quadrature, i.e., we look at $E^{\lambda }(z^{-\beta })/\lambda ^{-\beta }$ for $\beta =0.5$. The theory from Theorem 3.7 predicts behavior of the form $e^{-\frac{\gamma }{\ln (\lambda )k}}$, i.e., the rate drops like $\ln (\lambda )$. In Fig. 2, we can see this behavior quite well. In comparison, using standard $\textrm{sinc}$ quadrature gives a $\lambda$-robust asymptotic rate, but only of order $\sqrt{\mathcal {N}_{\text {q}}}$.

### A 2d example

In order to confirm our theoretical findings in a more complex setting, we now look at a 2d model problem with more realistic data than a single eigenfunction. Namely, we work in the PDE-setting of Remark 1.2 using the unit square $\Omega =(0,1)^2$ and the standard Laplacian with Dirichlet boundary conditions. We focus on two cases: first we look at what happens if the initial condition does not satisfy any compatibility condition, i.e., $u_0 \notin {\mathbb {H}}^{2\rho }$ for $\rho \ge 1/4$. The second example is then taken such that the data is (almost) in the Gevrey-type class as required by Theorem 2.8 and Theorem 2.11. The inhomogenity in time is taken as $f(t):=\sin (t)u_0$, thus possessing analogous regularity properties. We computed the function at $t=0.1$.

For the discretization in space and of the convolution in time of (2.8), we consider the scheme presented in [26]. It is based on *hp*-finite elements for the Galerkin solver and a *hp*-quadrature on a geometric grid in time for the convolution. As it is shown there, such a scheme delivers exponential convergence with respect to the polynomial degree and the number of quadrature points. Since we are not interested in these kinds of discretization errors, we fixed these discretization parameters in order to give good accuracy and only focus on the error due to discretizing the functional calculus. Namely, we used 5 layers of geometric refinement towards the boundary and vertices and a polynomial degree of $p=12$.

Since the exact solution is not available, we computed a reference solution with high accuracy and compared our other approximations to it. The reference solution is computed by the DE1 scheme (as it outperformed the others) by using 8 additional quadrature points to the finest approximation present in the graph. As the DE1 scheme has finished convergence at this point, we can expect this to be a good approximation.

We start with the parabolic problem. The initial condition is given by

$$\begin{aligned} u_0(x,y):=\omega ^{-1} \exp \Big (-\frac{(x-0.5)^2}{\omega }\Big ) \exp \Big (-\frac{(y-0.5)^2}{\omega }\Big ). \end{aligned}$$

**Fig. 3:**
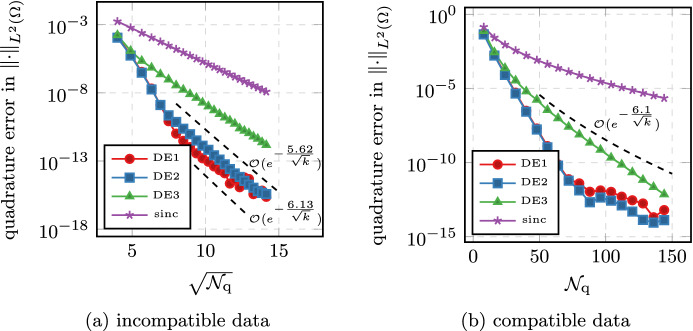
Comparison of quadrature schemes for 2d parabolic example; $\alpha =1/\sqrt{2}$, $\beta =0.7$

For $\omega :=1$, this function does not vanish near the boundary of $\Omega$ and therefore only satisfies $u_0 \in {\mathbb {H}}^{1/2-\varepsilon }$. We are in the setting of Lemma 2.10. By inserting $\rho =1/4$ (up to $\varepsilon$) and $r=0$, the predicted rates for DE1 and DE2 are roughly $e^{-\frac{6.13}{\sqrt{k}}}$ and $e^{-\frac{5.62}{k}}$ respectively. Figure 3a contains our findings. We observe that all methods converge with exponential rate proportional to $\sqrt{\mathcal {N}_{\text {q}}}$. The double exponential formulas outperforming the standard $\textrm{sinc}$ quadrature. We also observe that picking $\sigma \ne 1$ and $\theta \ne 1$ can greatly improve the convergence. The best results being delivered by DE1, i.e. $\sigma =1/2$ and $\theta =4$. For DE1 and DE2, we observe that for a large part of the computation, the scheme outperforms the predicted asymptotic rate, but for DE2, the rate appears sharp for large values of $\mathcal {N}_{\text {q}}$.

As a second example, we used $\omega =0.05$. This function is almost equal to 0 in a vicinity of the boundary of $\Omega$. Thus we may hope to achieve the improved convergence rate of Theorem 2.11. Figure 3b shows that it is plausible that the exponential rate of order $\mathcal {N}_{\text {q}}$ is achieved, and all the double exponential schemes greatly outperform the standard $\textrm{sinc}$ quadrature. The best results are again achieved by DE1 and DE2, which also greatly outperform the predicted rate for the non-smooth case.

### Elliptic problem and behavior for small $\beta$

Thus far, all our estimates worked under the assumption of $\beta>\overline{\beta }>0$. In order to shed some light on the behavior, and in addition gain insight into the behavior for the elliptic problem (2.4), we look at the following model problem for different values of $\beta$.

As geometry we again used the unit square. We chose $f=1$ as the constant function. In this class, we also included the method based on the Balakrishnan formula as well as a rational approximation method, namely the one based on computing the best uniform rational approximation as described in [17]. Where we approximate $z^{1-\beta }$ on [0, 1] using a rational function and then divide by $z^{-1}$ and scale back to the interval $[\lambda _{\min },\lambda _{\max }]$. For computing the approximation we used the brasil algorithm described in [18], the implementation of which can be found in the baryrat python package [18]. To determine $\lambda _{\max }$, we used a simple power iteration with 10 iterations. This gave the estimate $\lambda _{\max }\approx 6\cdot 10^{15}$. For $\lambda _{\min }$ we used the constant $\kappa :=3$ also used in the other algorithms.

For small $\beta$, preliminary experiments suggest a severe degrading of performance if the choice $k:=0.9 \ln (\mathcal {N}_{\text {q}})/\mathcal {N}_{\text {q}}$ is made. Therefore it was necessary to introduce the constant *r* in our considerations. We point out that setting $r:=1$ for $\beta >0.4$ is not fully necessary and only gives small improvements for larger values of $\beta$. Thus, if multiple values of $\beta$ are of interest, in order to be able to reuse the approximate inverses $(\mathcal {L}-\psi _{\sigma ,\theta }(j k))^{-1}$, the choice of this damping factor should be according to the smallest value of $\beta$ one is interested in.

**Fig. 4:**
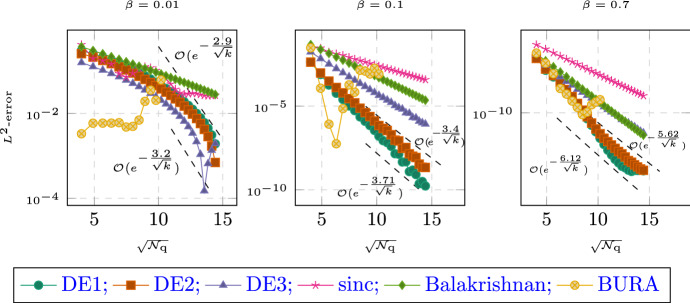
Comparison of approximation schemes for 2d elliptic problems with different parameter $\beta$

In Fig. 4, we again observe that with $\theta =4$ and $\sigma \in \{0.5,1\}$, the double exponential formulas significantly outperform the standard $\textrm{sinc}$ based strategies, where $\sigma =0.5$ again delivers the best performance. For comparison, we included the predicted rates for the DE1 and DE2 schemes into the graphics. We observe that asymptotically our estimates appear sharp, but with a large range of values, for which the scheme outperforms the predictions. The rational approximation method performs very well for small numbers of systems, but the performance degrades severely when higher accuracy is required. This instability with respect to numerical errors is most likely due to the requirement of rewriting the rational function in the partial fraction form to apply it to a matrix as described in [17] – even though a multiprecision library is used for computing the poles and residuals of the rational function. We also tried the method based on the AAA-algorithm [30], but there the numerical instability was even more problematic.

## Figures and Tables

**Fig. 5:**
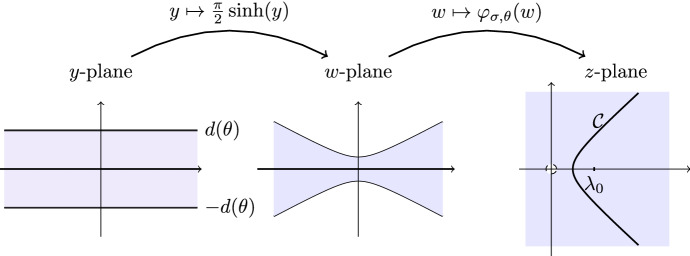
Illustration of the different planes involved in the mapping $\psi _{\sigma ,\theta }$

## Appendix A: Properties of the coordinate transform $\psi _{\sigma ,\theta }$

In this appendix, we study the transformation $\psi _{\sigma ,\theta }$ in detail, as it is crucial to understand the double exponential quadrature scheme. Since this transformation is itself defined in a two-part way, we introduce the following nomenclature.

### Definition A.1

We call the complex plane on which $\psi _{\sigma ,\theta }$ is defined the *y*-*plane*, mainly using the parameter *y* for its points. The main subset of interest there is the strip $D_{d(\theta )}$.

Using the function $y \mapsto \frac{\pi }{2}\sinh (y)$, the *y*-plane is mapped to the *w*-*plane*. The most interesting set is the image of $D_{d(\theta )}$ under this deformation, denoted by $H_\theta :=\big \{\frac{\pi }{2}\sinh (y), y\in D_{d(\theta )}\big \}$ (named due to its hyperbola shape).

Finally, using the function $\varphi _{\sigma ,\theta }(w):=\kappa [\cosh (\sigma w)+i\theta \sinh (w)]$, mapping $H_\theta \rightarrow \mathbb {C}$, we arrive at the *z*-*plane* which corresponds to the range of $\psi _{\sigma ,\theta }$, and the domain of the functions used for the Riesz–Dunford calculus. The situation is summarized in Fig. 5.

If we talk about generic complex numbers without relation to any of the specific planes, we use the letter $\zeta$ instead.

We start out with some basic properties of $\sinh$.

### Lemma A.2

The map $y \mapsto \frac{\pi }{2} \sinh (y)$ has the following properties:

(i) It is a bijective mapping $D_{d(\theta )}\rightarrow H_\theta$, see Fig. 5.
(ii) For $\left| {\text {Re}}(\zeta )\right| \ge \zeta _0 > 0$, there exist constants $c_1, c_2>0$ depending only on $\zeta _0$ such that
  $$\begin{aligned} c_1\left| \sinh (\zeta )\right| \le \left| \cosh (\zeta )\right| \le c_2 \left| \sinh (\zeta )\right| . \end{aligned}$$
(iii) For any $\delta < \pi /2$, $\sinh$ maps the domain $D^{\textrm{exp}}_\delta$, as defined in (3.1), to a strip of size $\delta$, i.e.,
  A1 $$\begin{aligned} \left| {\text {Im}}(\sinh (y))\right| <\, \delta \qquad \forall y \in D^{\textrm{exp}}_\delta . \end{aligned}$$

### Proof

*Proof of* (i): It is well known that $\sinh$ is injective for $\left| {\text {Im}}(y)\right| <\pi /2$. Since $H_\theta$ is defined as the range of the map this is sufficient.

Part (ii) is an easy consequence of the fact that $\sinh$ and $\cosh$ have the same asymptotic behavior for ${\text {Re}}(\zeta )\rightarrow \infty$. To see (iii), we estimate for $y \in D^{\textrm{exp}}_\delta$:

$$\begin{aligned} \left| {\text {Im}}(\sinh (y))\right|&=\cosh ({\text {Re}}(y)) \left| \sin ({\text {Im}}(y))\right| <\, \delta \,\cosh ({\text {Re}}(y)) e^{-\left| {\text {Re}}(y)\right| } \le \delta . \end{aligned}$$

$$\square $$

### Lemma A.3

$\psi _{\sigma ,\theta }$ is analytic on the infinite strip $D_{d(\theta )}$. For $d(\theta )$ sufficiently small, both $\psi _{\sigma ,\theta }$ and $\psi _{\sigma ,\theta }'$ are non-vanishing on $D_{d(\theta )}$.

### Proof

The analyticity of $\psi _{\sigma ,\theta }$ is clear. In order to analyze the roots, we first rewrite for $w=a+ib$, separating the real and imaginary parts:

A2 $$\begin{aligned} \begin{aligned} \cosh (\sigma w)+i\theta \sinh (w)&=\Big (\cosh (\sigma a)\cos (\sigma b)-\theta \cosh (a) \sin (b) \Big )\\&\quad \ + i\Big (\sinh (\sigma a)\sin (\sigma b)+\theta \sinh (a)\cos (b)\Big ). \end{aligned} \end{aligned}$$

We first focus on **the case** $\varvec{\sigma }\mathbf {=1}$. In this case,  (A2) shows that any root *y* of $\psi _{\sigma ,\theta }$ must satisfy for $w:=\frac{\pi }{2}\sinh (y)=:a+bi$:

$$\begin{aligned} \cosh (a) \Big (\cos (b)-\theta \sin (b)\Big )&=0 \quad \text { and } \quad \sinh (a) \Big (\theta \cos (b)+\sin (b)\Big ) = 0. \end{aligned}$$

Since $\cosh$ has no roots, we get $\cos (b)=\theta \sin (b)$. As $\cos (b)=\theta \sin (b)$ and $\theta \cos (b)=-\sin (b)$ is impossible at the same time, we get that $a=0$ and $b=\tan ^{-1}(1/\theta ) + \ell \pi$ for some $\ell \in \mathbb {Z}$.

It remains to show that $\frac{\pi }{2}\sinh (y)$ does not map to these points. Looking at the real part of $\sinh (y)$ we immediately deduce that if ${\text {Im}}(y) \in (-\pi /2,\pi /2)$, in order to produce a purely imaginary result, it must hold that ${\text {Re}}(y)=0$. For the imaginary part, we then get the equation:

$$\begin{aligned} \sin ({\text {Im}}(y)) = 2\ell +\frac{2\tan ^{-1}(1/\theta )}{\pi } \quad \text {for some}\, \ell \in \mathbb {Z}\end{aligned}$$

which is not possible for $\left| {\text {Im}}(y)\right| \le d(\theta ) < \sin ^{-1}(\frac{2\tan ^{-1}(1/\theta )}{\pi })$. Next, we show that $\psi _{\sigma ,\theta }'$ also does not vanish. A simple calculation shows

A3 $$\begin{aligned} \psi _{1,\theta }'(y)=i\theta \,\psi _{1,\frac{1}{\theta }}(-y)\frac{\pi }{2}\cosh (y). \end{aligned}$$

Since the restriction $\theta \ge 1$ was not crucial for the proof, $\psi _{1,\frac{1}{\theta }}$ and $\cosh$ have no roots in the symmetric (w.r.t. sign flip) domain $D_{d(\theta )}$. This shows that $\psi _{1,\theta }'$ also is non-vanishing.

**The case** $\mathbf {\varvec{\sigma }=1/2}$ is similar, but a little more involved. We first show that all zeros of $\cosh (\sigma w) +i\theta \sinh (w)$ satisfy ${\text {Re}}(w)=0$ and $\left| {\text {Im}}(w)\right|> w_0 >0$ for a constant $w_0$ depending only on $\theta$. If $\cosh (w/2)\ne 0$ we can use the double angle formula for $\sinh$ to get

$$\begin{aligned} 0=\cosh (w/2)\big (1 + 2\theta i\sinh (w/2)\big ) \qquad \text{ which } \text{ implies } \qquad \sinh (w/2) = \frac{i}{2\theta }. \end{aligned}$$

Splitting into real and imaginary part, we get for $w=a+ib$:

$$\begin{aligned} \sinh (a/2)\cos (b/2) =0 \qquad \text{ and } \qquad \cosh (a/2)\sin (b/2) = \frac{1}{2\theta }. \end{aligned}$$

If $a\ne 0$, we get that $\cos (b/2)=0$ and thus $\sin (b/2)= \pm 1$. This would imply that $\left| \cosh (a/2)\right| =\frac{1}{2\theta } \le 1/2$ which is a contradiction. If $a=0$, we get that

$$\begin{aligned} \left| b\right| \ge 2 \big |{\sin ^{-1}\Big (\frac{1}{2\theta }\Big )}\big |> 0. \end{aligned}$$

Similarly, one can argue if $\cosh (w/2)=0$, that $a=0$ and $b=\pi (2\ell +1)$. We then proceed just like in the case $\sigma =1$ to conclude that $\frac{\pi }{2}\sinh$ does not map to such points.

In order to investigate its roots, we compute the derivative of $\psi _{\frac{1}{2},\theta }$ as

A4 $$\begin{aligned} \psi _{\frac{1}{2},\theta }'(y)&=\Big (\frac{1}{2}\sinh \big (\pi \sinh (y)/4\big ) + i\theta \cosh \big (\pi \sinh (y)/2\big )\Big )\frac{\pi }{2}\cosh (y). \end{aligned}$$

Our main concern is when the first bracket reaches zero. Substituting $t:=\sinh (\pi \sinh (y)/4)$ and using the double angle formula for $\cosh$ we get

$$\begin{aligned} 0&=\frac{t}{2} + i\theta (1+2t^2). \end{aligned}$$

Solving this equation, we get that *t* is purely imaginary and for $\theta \ge 1$ satisfies $0<\left| {\text {Im}}(t)\right| <1$. Again writing $w=:a+ib$ we get

$$\begin{aligned} \sinh (a/2)\cos (b/2) =0 \qquad \text{ and } \qquad \cosh (a/2)\sin (b/2) = {\text {Im}}(t). \end{aligned}$$

Just like we did when showing $\psi _{\frac{1}{2},\theta }\ne 0$ we can argue that $a=0$. We get $\sin (b/2)={\text {Im}}(t)$. Since *t* only depends on $\theta$, we get that $\left| b\right|> b_0 > 0$ with a constant only depending on $\theta$. We proceed as when showing $\psi _{\frac{1}{2},\theta }\ne 0$ to conclude that $\psi _{\frac{1}{2},\theta }'$ has no root in $D_{d(\theta )}$ for *d* sufficiently small (depending on $\theta$). $\square$

Next, we study the growth of $\psi _{\sigma ,\theta }(y)$ as $\left| {\text {Re}}(y)\right| \rightarrow \infty$.

### Lemma A.4

Assume that $d(\theta )<1/2$. Then there exist constants $c_1, c_2$, $\gamma _1, \gamma _2 >0$ such that for $y \in D_{d(\theta )}$ we can estimate

A5 $$\begin{aligned} c_1 \exp (\gamma _1 e^{\left| {\text {Re}}(y)\right| }) \le \left| \psi _{\sigma ,\theta }(y)\right| \le c_2 \exp (\gamma _2 e^{\left| {\text {Re}}(y)\right| }), \end{aligned}$$

i.e., the growth of $\psi$ is double exponential. Additionally, we can bound

A6 $$\begin{aligned} |{\psi _{\sigma ,\theta }'}(y)|\lesssim |\psi _{\sigma ,\theta }(y)|\cosh ({\text {Re}}(y)). \end{aligned}$$

### Proof

We start with the simple case $\varvec{\sigma }\mathbf {=1}$, and focus on what happens if $\left| {\text {Re}}(y)\right|> y_0>0$ for a to be tweaked constant $y_0$. The values with $0\le \left| {\text {Re}}(y)\right| \le y_0$ can be covered by adjusting $c_1$ and $c_2$, due to the compactness of the set $\{y \in D_{d(\theta )}: \left| {\text {Re}}(y)\right| \le y_0\}$ and the fact that $\left| \psi _{\sigma ,\theta }(y)\right| >0$ by Lemma A.3. We first only consider the $y-w$ part of the transformation. We compute, since $\frac{1}{2}<\cos (t)\le 1$ for $t \in (-1/2,1/2)$:

$$\begin{aligned} \left| {\text {Re}}(\sinh (y))\right|&= \left| \sinh ({\text {Re}}(y))\right| \cos ({\text {Im}}(y)) \sim \left| \sinh ({\text {Re}}(y))\right| \sim c_1 e^{\left| {\text {Re}}(y)\right| }. \end{aligned}$$

Easy calculation shows that for $h_1(\eta ):=\left| \cos (\eta ) - \theta \sin (\eta )\right|$ and $h_2(\eta ):=\left| \theta \cos (\eta ) + \sin (\eta )\right|$ it holds that $[h_1(\eta )]^2+[h_2(\eta )]^2=1+\theta ^2$. Thus, for $w \in H_\theta$ with $\left| {\text {Re}}(w)\right| \ge 1$, we can calculate:

A7 $$\begin{aligned} \left| \cosh (w) + i\theta \sinh (w)\right| ^2= & {} \left| \cosh ({\text {Re}}(w))\right| ^2[h_1({\text {Im}}(w))]^2 \nonumber \\{} & {} + \left| \sinh ({\text {Re}}(w))\right| ^2 [h_2({\text {Im}}(w))]^2 \nonumber \\ > rsim & {} \min \big (\cosh ({\text {Re}}(w)),\left| \sinh ({\text {Re}}(w))\right| \big )^2(1+\theta ^2) \;\nonumber \\ > rsim & {} \; e^{2\left| {\text {Re}}(w)\right| }. \end{aligned}$$

Overall, we see the lower bound of (A5). The upper bound is easily seen, as $\left| \sinh (y)\right|$ and $\left| \cosh (y)\right|$ both grow exponentially and the bound only depends on the real part of the argument. (A6) follows from (A3) and the asymptotics (A7)and (A5).

We now look at how to adapt the proof to **the case** $\varvec{\sigma }\mathbf {=1/2}$. If $\left| {\text {Re}}(w)\right| \ge 2\ln (1+\sqrt{5})$, we get

A8 $$\begin{aligned}{} & {} \left| \cosh (w/2) + i\theta \sinh (w)\right| \nonumber \\{} & {} \quad \ge \theta \left| \sinh (w)\right| - \left| \cosh (w/2)\right| \ge \frac{1}{2}\Big (e^{\left| {\text {Re}}(w)\right| } - 2 - e^{\left| {\text {Re}}(w)/2\right| }\Big ) \ge \frac{1}{4} e^{\left| {\text {Re}}(w)\right| },\qquad \quad \end{aligned}$$

where in the last step we used the monotonicity of the expression and the fact that $\frac{e^{\left| {\text {Re}}(w)\right| }}{2} - e^{\left| {\text {Re}}(w)/2\right| }=2$ for ${\text {Re}}(w)=2\ln (1+\sqrt{5})$. The argument for the $y-w$-transformation stays the same. The upper bound also follows easily from the triangle inequality and the growth of $\sinh$ and $\cosh$.

To see (A6), we combine (A4) with the asymptotic estimate (A8) to get

$$\begin{aligned} |{\psi _{\sigma ,\theta }'}(y)|&\lesssim e^{\pi \left| {\text {Re}}(\sinh (y))\right| /2} \cosh ({\text {Re}}(y)) \lesssim |\psi _{\sigma ,\theta }(y)|\cosh ({\text {Re}}(y)). \end{aligned}$$

$$\square $$

While on the full strip $D_{d(\theta )}$, the image of the transformation is difficult to study, the restriction to a certain subdomain is much better behaved.

### Lemma A.5

For $\sigma =1$, there exists a constant $\delta >0$, depending on $\theta$, such that restricted to the domain $D^{\textrm{exp}}_\delta$, $\psi _{1,\theta }$ maps to a sector in the right-half plane,

$$\begin{aligned} S_{\omega }:=\big \{z \in \mathbb {C}: \left| {\text {Arg}}(z)\right| \le \omega \big \} \qquad \text {with} \quad \omega <\frac{\pi }{2}. \end{aligned}$$

For $\sigma =1/2$, and for all $\varepsilon >0$, there exists a constant $\delta >0$, depending on $\theta$ and $\varepsilon$, such that restricted to the domain $D^{\textrm{exp}}_\delta$ the transformation $\psi _{\frac{1}{2},\theta }$ maps to the sector $S_{\pi /2+\varepsilon }$.

In both cases, there exist constants $c_1, c_2>0$ such that $\psi _{\sigma ,\theta }$ satisfies for all $\lambda \ge \lambda _0 > \kappa :$

A9 $$\begin{aligned} \left| \psi _{\sigma ,\theta }(y)-\lambda \right| \ge c_1 \; \text {and}\; \left| \psi _{\sigma ,\theta }'(y)(\psi _{\sigma ,\theta }(y) - \lambda )^{-1}\right| \le c_2 \cosh ({\text {Re}}(y)) \quad \forall y \in D^{\textrm{exp}}_\delta , \end{aligned}$$

where $c_1, c_2$ only depend on $\lambda _0$ and $\theta$.

### Proof

By Lemma A.2(iii) it is sufficient to consider the mapping of $\varphi _{\sigma ,\theta }$ restricted to small strips in the *w*-plane around the real axis. We start with the simpler **case** $\varvec{\sigma }\mathbf {=1}$. Going back to (A2) and writing $w:=\frac{\pi }{2} \sinh (y)=:a+ib$, we note that if $\left| b\right|$ is sufficiently small, we can guarantee that ${\cos (b)- \theta \sin (b)}> c >0$ for some constant $c >0$ depending on $\theta$.

We have

$$\begin{aligned} {\text {Re}}(\varphi _{\sigma ,\theta }(w))&=\kappa \cosh (a)\big (\cos (b)-\theta \sin (b)\Big ) > c \, \kappa \cosh (a), \\ \left| {\text {Im}}(\varphi _{\sigma ,\theta }(w))\right|&=\kappa \left| \sinh (a)\right| |\theta \cos (b)+ \sin (b)| \le (1+\theta ) \kappa \left| \sinh (a)\right| . \end{aligned}$$

This implies

$$\begin{aligned} 0\le \frac{\left| {\text {Im}}(\varphi _{\sigma ,\theta }(w))\right| }{{\text {Re}}(\varphi _{\sigma ,\theta }(w))}\le (1+\theta ) c^{-1} \qquad \forall w\in \mathbb {C},\; \left| {\text {Im}}(w)\right| \text {sufficiently small}. \end{aligned}$$

Next, we show that **for** $\varvec{\sigma =1/2}$, sufficiently thin strips in the *w*-domain are mapped to sectors with opening angle $\pi /2+\varepsilon$. Such sectors are characterized by

$$\begin{aligned} -{\text {Re}}(\varphi _{\sigma ,\theta }(w)) \le \widetilde{\varepsilon } \left| {\text {Im}}(\varphi _{\sigma ,\theta }(w))\right| \qquad \forall \left| {\text {Im}}(w)\right| <b_0(\widetilde{\varepsilon }) \end{aligned}$$

for $\widetilde{\varepsilon } > 0$ depending on $\varepsilon$. The interesting case is ${\text {Re}}(\varphi _{\sigma ,\theta }(w))<0$. There, we get for $\left| b\right| \le \pi$:

$$\begin{aligned} -{{\text {Re}}(\varphi _{\sigma ,\theta }(w))}&=-\kappa \cosh (a/2)\cos (b/2)+\kappa \theta \cosh (a) \sin (b) \le \kappa \theta \cosh (a) \left| \sin (b)\right| . \end{aligned}$$

For the imaginary part, the double angle-formula gives:

$$\begin{aligned} \left| {\text {Im}}(\varphi _{\sigma ,\theta }(w))\right|&\ge \kappa \theta \left| \sinh (a) \cos (b)\right| - \kappa \left| \sinh (a/2)\sin (b/2)\right| \\&\ge \frac{\kappa \theta }{2} \left| \sinh (a) \cos (b)\right| + \kappa \left| \sinh (a/2)\right| \big (\theta \cosh (a/2)\left| \cos (b)\right| - \left| \sin (b/2)\right| \big ). \end{aligned}$$

For $\left| b\right|$ sufficiently small, we get $\theta \cos (b) - \left| \sin (b/2)\right| >0$, and thus the last term is non-negative. We conclude

$$\begin{aligned} -{{\text {Re}}(\varphi _{\sigma ,\theta }(w))}&\le 2\left| \frac{\cosh (a)}{\sinh (a)} \frac{\sin (b)}{\cos (b)}\right| \left| {\text {Im}}(\varphi _{\sigma ,\theta }(w))\right| . \end{aligned}$$

For $a>1$, the ratio  is uniformly bounded. By making *b* sufficiently small, we can ensure $\left| \sin (b)\right| /{\cos {b}}< \widetilde{\varepsilon }$. For $a<1$, we note that by restricting the values of *b* it can be easily seen that ${\text {Re}}(\varphi _{\sigma ,\theta }(w))\ge 0$. Thus we can conclude that $\psi _{\sigma ,\theta }$ maps to the stated sectors.

Next, we prove the bounds on the distance to the real axis, again primarily investigating the behavior of $\varphi _{\sigma ,\theta }$ in thin strips. We focus on $\varvec{\sigma =1/2}$. Since $\varphi _{\sigma ,\theta }(0)=\kappa < \lambda _0$ and $\varphi _{\sigma ,\theta }$ is continuous, there exist constants $0<q<1$ and $\widetilde{\delta }>0$ such that $\left| \varphi _{\sigma ,\theta }(w) - \varphi _{\sigma ,\theta }(0)\right| < q(\lambda _0-\kappa )$ for all $\left| w\right| <\widetilde{\delta }$. This gives:

$$\begin{aligned} \left| \varphi _{\sigma ,\theta }(w)-\lambda \right|&\ge \lambda - \left| \varphi _{\sigma ,\theta }(w)\right| \ge \lambda _0 -\left| \varphi _{\sigma ,\theta }(0)\right| - \left| \varphi _{\sigma ,\theta }(w)-\varphi _{\sigma ,\theta }(0)\right| \\&>\lambda _0 - \kappa - q (\lambda _0 - \kappa ) \ge (1-q) (\lambda _0 - \kappa )>0 \qquad \qquad \forall \left| w\right| \le \widetilde{\delta }. \end{aligned}$$

By selecting $\delta < \widetilde{\delta }/2$ in the definition of $D^{\textrm{exp}}_\delta$, we may then continue by only considering $\left| a\right| :=\left| {\text {Re}}(w)\right| > \widetilde{\delta }/2$. The imaginary part of $\varphi _{\frac{1}{2},\theta }(y)$ satisfies:

$$\begin{aligned} {\text {Im}}(\varphi _{\frac{1}{2},\theta }(y))&=\kappa \sinh (a/2)\sin (b/2)+\kappa \theta \sinh (a)\cos (b) \\&= \kappa \sinh (a/2)\Big (\sin (b/2) + 2\theta \cosh (a/2) \cos (b) \Big ). \end{aligned}$$

For $\left| b\right| \le \pi /4$ we have $\sin (b/2) + 2\cos (b) > 0$ and thus can conclude that $|{{\text {Im}}(\varphi _{\frac{1}{2},\theta }(y))}| \ge c > 0$ and in turn $|{\varphi _{\frac{1}{2},\theta }(y) - \lambda }| \ge c > 0$. The case $\sigma =1$ follows similarly, but not using the double angle formula.

To see that $\psi _{\sigma ,\theta }'(y)(\psi _{\sigma ,\theta }(y)-\lambda )^{-1}$ can also be uniformly bounded, we only need to focus on large values of *y* (and therefore *w*). Asymptotically, we estimate for $\left| b\right| <\pi /4$:

$$\begin{aligned} \left| {\text {Im}}(\psi _{\sigma ,\theta }(y))\right|&\ge -\kappa \left| \sinh (\sigma a)\right| \left| \sin (\sigma b)\right| +\kappa \theta \left| \sinh (a)\cos (b)\right| > rsim \left| \sinh (a)\right| \\&\!\!{\mathop { > rsim }\limits ^{\text {(A7) or (A8)}}} \!\! \left| \psi _{\sigma ,\theta }(y)\right| . \end{aligned}$$

Using (A6) then concludes the proof. $\square$

In order to apply the double exponential formulas for the Riesz–Dunford calculus, it is important to understand where $\psi _{\sigma ,\theta }(z)$ hits the real line. We start with the *w*-domain.

### Lemma A.6

Fix $\lambda \ge \lambda _0 > 1$. Then the following holds for every $w\in \mathbb {C}$ with ${\text {Re}}(w)\ne 0$ and

A10 $$\begin{aligned} \cosh (\sigma w) + i\theta \sinh (w) = \lambda : \end{aligned}$$

(i) There exist constants $c_1,c_2,c_3>0$ such that *w* satisfies $\displaystyle \log (\lambda ) - c_1 \le \left| {\text {Re}}(w)\right| \le \log (\lambda ) + c_1$ and
  $$\begin{aligned} \left| {\text {Im}}(w)\right| \ge {\left\{ \begin{array}{ll} \tan ^{-1}(\theta ) &{} \text {if }\sigma =1 \\ \max \big (\frac{\pi }{2} - \frac{c_2}{\theta \sqrt{\lambda }},c_3) &{} \text {if } \sigma =1/2 \end{array}\right. }, \end{aligned}$$
  where $c_1$ depends on $\lambda _0$ and $\theta$, $c_2$ depends on $\lambda _0$, and $c_3$ depends on $\theta$.
(ii) Given $0<r<R$, the number $N_w(\lambda ,r, R)$ of points *w* satisfying (A10) with $r\le \left| {\text {Im}}(w)\right| \le R$ is bounded uniformly in $\lambda$ by
  $$\begin{aligned} N_w(\lambda ,r,R) \le C \left| R-r\right| \end{aligned}$$
  The constant *C* depends only on $\theta$.
(iii) There exist at most four values $p_1,\dots ,p_4$ depending on $\lambda$, $\theta$, and $\sigma$ such that all points satisfying (A10) can be written as
  A11 $$\begin{aligned} w=p_j + \frac{2\ell }{\sigma } \pi i\qquad \text { for some}\, \ell \in \mathbb {Z}, j\in 1, \dots , 4. \end{aligned}$$
  If *w* solves (A10) then $-\overline{w}$ does as well.

### Proof

We start with the simpler case $\sigma =1$. By separating the real and imaginary parts as in (A2), we can observe that the critical points $w=a+ib$ with $a\ne 0$ are located at

A12 $$\begin{aligned} \cosh (a)&= \frac{\lambda }{\sqrt{1+\theta ^2}}, \qquad b=-\tan ^{-1}(\theta ) + 2\ell \pi , \quad \ell \in \mathbb {Z}. \end{aligned}$$

This implies that $\left| a\right| \sim \ln (\lambda )$, and we also see that for each $\ell$, there are at most two such points, one in each half-plane. All the statements follow easily. Note that in (iii) only two families are needed.

For the remainder of the proof we therefore focus on the case $\sigma =1/2$. **Proof of **(i): We start with the bound on the real part and write $w=a+ib$. We note that if $\left| a\right| > \max \big (1,2\ln \big (\frac{8}{ \theta }\big )\big )$ one can estimate using elementary considerations that $e^{\left| a\right| }/4 \le \left| \sinh (w)\right|$ and $e^{\left| a\right| /2}/\theta \le e^{\left| a\right| }/8$.

We then calculate:

$$\begin{aligned} \frac{e^{\left| a\right| }}{4}&\le \left| \sinh (w)\right| =\frac{1}{\theta } {\left| \lambda - \cosh (w/2)\right| } \le \frac{\lambda }{\theta } + \frac{e^{\frac{\left| a\right| }{2}}}{\theta } \le \frac{\lambda }{\theta } + \frac{e^{\left| a\right| }}{8}. \end{aligned}$$

From this, the statement readily follows. The other direction is shown similarly:

$$\begin{aligned} e^{|a|}&\ge \left| \sinh (w)\right| =\frac{1}{\theta } {\left| \lambda - \cosh (w/2)\right| } \ge \frac{\lambda }{\theta } - \frac{e^{\frac{\left| a\right| }{2}}}{\theta } \ge \frac{\lambda }{\theta } - \frac{e^{\left| a\right| }}{8}. \end{aligned}$$

For $\left| a\right| \le \max \big (1,2\ln \big (\frac{8}{ \theta }\big )\big )$, we use the bound $\left| \varphi _{\sigma ,\theta }(w)\right| \lesssim e^{\left| a\right| }$ to see that $\lambda =\varphi _{\sigma ,\theta }(w) \lesssim e^{\left| a\right| }$, giving that $\ln (\lambda )$ must be uniformly bounded. By taking $c_1$ large enough we can make the $\ln (\lambda )-c_1$ negative, thus making the first estimate in (i) trivial. Since $\ln (\lambda )\ge \ln (\lambda _0)> 0$, we can also immediately see

The final bound on the real part of *w* then follows for $c_1:=\max \big (\ln (8/\theta ),\ln (9\theta /8),\widetilde{c}\big )$, where $\widetilde{c}$ is used to compensate for the case of small *a*.

Looking at the imaginary part of equation (A10), we get from the double-angle formulas

A13 $$\begin{aligned} 0&=\sinh (a/2)\sin (b/2) + \theta \sinh (a)\cos (b) \nonumber \\&=\sinh (a/2)\Big (\!\sin (b/2)+2\theta \cosh (a/2) \big (1-2\sin ^2(b/2)\big )\!\Big ). \end{aligned}$$

Since we assume $a\ne 0$, we get by substituting $\tau :=\sin (b/2)$

A14 $$\begin{aligned} 0=\tau +2\theta \cosh (a/2) (1-2\tau ^2) \qquad \text { or }\qquad \tau =\frac{1 \pm \sqrt{1+32 \theta ^2 \cosh ^2(a/2)}}{8 \theta \cosh (a/2)}. \end{aligned}$$

From this, using the asymptotic behavior

$$\begin{aligned} \tau = \pm \frac{1}{\sqrt{2}} + \mathcal {O}\Big (\frac{1}{\theta \cosh (a/2)}\Big ) \quad \text { and } \quad \sin ^{-1}\Big (\frac{1}{\sqrt{2}} + h\Big ) = \frac{\pi }{4} + \mathcal {O}(h) \end{aligned}$$

the statement follows for large *a*, since $b=2\sin ^{-1}(\tau )$ and $\cosh (a/2) > rsim \sqrt{\lambda }$ by (i). For small *a*, we note that (A14) shows that ${\text {Im}}(w)\ne 0$. By continuity, it must therefore hold that $\left| {\text {Im}}(w)\right| >0$ uniformly.

**Proof of** (ii) and (iii): We square the defining equation (A10), getting

A15 $$\begin{aligned} \lambda ^2&=\cosh ^2(w/2)+2i\theta \cosh (w/2)\sinh (w)-\theta ^2 \sinh ^2(w) \nonumber \\&=\cosh ^2(w/2)+4i\theta \cosh ^2(w/2)\sinh (w/2)-4\theta ^2 \sinh ^2(w/2)\cosh ^2(w/2). \end{aligned}$$

Writing $\cosh ^2(w/2)=1+\sinh ^2(w/2)$, we get that $t:=\sinh (w/2)$ solves the quartic equation

A16 $$\begin{aligned} \lambda ^2&=1+t^2+4i\theta (1+t^2)t-4\theta ^2 t^2 - 4\theta ^2 t^4. \end{aligned}$$

This means there can be at most 4 such values $t_{1}, \dots t_4$ for any $\lambda$ and it must hold that

A17 $$\begin{aligned} \sinh (w/2)&=t_j \quad \text {or } \quad w=w_j + 4\pi \ell i\qquad \ell \in \mathbb {Z}\end{aligned}$$

Here $w_j$ for $j=1,\dots ,4$ is the solution to $\sinh (w_j/2)=t_j$ with ${\text {Re}}(w_j)>0$ and minimal value of $\left| {\text {Im}}(w_j)\right|$.

To see (ii), we note that for each $t_j$ at most  values lie in the sought after strip. Therefore we can estimate

The statement (iii) follows readily from (A17). The fact that $-\overline{w_j}$ also solves (A10) follows by conjugating both sides of the Eq. (A16).

$$\square $$

Next, we show that points which have positive distance to the poles in the *w*-plane are mapped to points with distance $\lambda$ in the *z*-plane. Note that in the following Lemma we include some additional points in order to avoid distinguishing more cases. We also exclude most of the imaginary axis, as for small values of $\lambda$ it might contain poles which are structurally different than the ones involving large $\lambda$.

### Lemma A.7

Define

$$\begin{aligned} b_0:=\max \big \{b\ge 0: \quad \left| \varphi _{\sigma ,\theta }(i\tau )\right| \le (\lambda _0 + \kappa )/2 \quad \forall \left| \tau \right| \le b\big \}. \end{aligned}$$

Fix $\lambda \ge \lambda _0 > \kappa$ and define the set

$$\begin{aligned} {\mathcal {M}}_{\lambda }&:=\big \{ p_\lambda + i \ell \pi , \, \text {with }\; p_\lambda \in \mathbb {C}\text { such that } \varphi _{\sigma ,\theta }(p_\lambda ) = \lambda \text { and }\ell \in \mathbb {Z}\big \} \cup \{ ib : \, \left| b\right| \le b_0 \}. \end{aligned}$$

For any $\delta > 0$, there exists a constant $c(\delta )>0$, depending only on $\delta$ and $\theta$, such that for all $w \in \mathbb {C}$ with ${\text {dist}}(w,{\mathcal {M}}_\lambda )> \delta$ we can estimate

$$\begin{aligned} \left| \varphi _{\sigma ,\theta }(w) - \lambda \right| \ge c(\delta ) \lambda . \end{aligned}$$

### Proof

Without loss of generality, we assume $\delta < b_0$. We first deal with the issues close to the imaginary axis. Since $\left| \varphi _{\sigma ,\theta }(ib)\right| \le (\lambda _0+\kappa )/2$ if $\left| b\right| \le b_0$, we can find $\varepsilon \in (0,\delta /2)$ such that

$$\begin{aligned} \left| \varphi _{\sigma ,\theta }(\widetilde{w})\right| < 3\lambda _0/4 + \kappa /4 \quad \text {for all} \quad \widetilde{w} \in U_{\varepsilon }&:=\big \{ \left| {\text {Re}}(\widetilde{w})\right| \le \varepsilon \; \text {and}\;\left| {\text {Im}}(\widetilde{w})\right| \le b_0 \big \}. \end{aligned}$$

If $\left| {\text {Re}}(w)\right| \le \varepsilon$, the distance condition to ${\mathcal {M}}_{\lambda }$ implies for all $\left| b\right| \ge b_0$:

$$\begin{aligned} \delta ^2 \le |w- ib|^2 = |{\text {Im}}(w) - b|^2 + |{\text {Re}}(w)|^2 \le |{\text {Im}}(w) - b|^2 + \delta ^2/4, \end{aligned}$$

or $|{\text {Im}}(w) - b| \ge \delta /2$. Since all $\left| b\right| \ge b_0$ are admissible, it can not be that $|{\text {Im}}(w)| \ge b_0$. Therefore we readily see $\left| {\text {Im}}(w)\right| \le b_0 - \delta /2$ and thus $w \in U_{\varepsilon }$.

We can therefore calculate:

$$\begin{aligned} \left| \varphi _{\sigma ,\theta }(w)-\lambda \right| \ge \lambda - \left| \varphi _{\sigma ,\theta }(w)\right| \ge \frac{\lambda }{4}\Big (1 - \frac{\kappa }{\lambda _0}\Big ). \end{aligned}$$

Next, we deal with small values of $\lambda$. For constants $\Lambda$, $C_w$ to be fixed later, consider $\lambda \in [\lambda _0, \Lambda ]$ and define the set

$$\begin{aligned} {\mathcal {R}}:=\{w \in \mathbb {C}: \; \left| {\text {Re}}(w)\right| \ge \varepsilon , \left| w\right| \le C_w\}. \end{aligned}$$

We show that the stated bound holds for $w \in {\mathcal {R}}$. By Lemma A.6(iii), the points of ${\mathcal {M}}_\lambda$ can be written as

$$\begin{aligned} {\mathcal {M}}_\lambda =\Big \{ p_{j} + \pi \ell i, \;\; \ell \in \mathbb {Z},\; j=1,\dots ,4 \Big \} \cup \Big \{ ib : \; \left| b\right| \le b_0 \Big \} \end{aligned}$$

for some reference points $p_{j} \in \mathbb {C}$, w.l.o.g. $\left| {\text {Im}}(p_j)\right| \le \pi$. We introduce set

A18 $$\begin{aligned} {\mathcal {M}}_{\Lambda ,C_w}:=\Big \{p_{j} + \ell \pi i, \; \left| \ell \right| \le L, \, j=1,\dots ,4 \Big \} \end{aligned}$$

where $L:=\lceil (C_w+1)/\pi \rceil$ is taken large enough that for all $\lambda \in [\lambda _0, \Lambda ]$, it holds that

$$\begin{aligned} {\mathcal {M}}_{\lambda } \cap {\mathcal {R}} \subseteq {\mathcal {M}}_{\Lambda ,C_w}, \end{aligned}$$

i.e., it contains all the poles of size less or equal than $C_w$ uniformly in $\lambda$ (but possibly also some larger ones). We consider the map

$$\begin{aligned} \Phi (\lambda , w):= \big (\varphi _{\sigma ,\theta }(w) - \lambda \big )^{-1}\prod _{p_\lambda \in {\mathcal {M}}_{\Lambda ,C_w}}{(w - p_\lambda )}. \end{aligned}$$

By the inverse function theorem, the points $p_{j}=\varphi _{\sigma ,\theta }^{-1}(\lambda )$ depend continuously on $\lambda$ since $\varphi _{\sigma ,\theta }'\ne 0$ away from the imaginary axis (where we stay away from by construction). Thus, also the other points $p_{\lambda }$ depend continuously on $\lambda$. Similarly, the denominator only has simple zeros for $w\in {\mathcal {M}}_\lambda$. Since, in that case the numerator also vanishes one can argue that $\Phi$ has a continuous extension to $[\lambda _0, \Lambda ]\times {\mathcal {R}}$ which is bounded, i.e., it holds

$$\begin{aligned} \left| \frac{\prod _{p_\lambda \in {\mathcal {M}}_{\Lambda ,C_w}}{(w - p_\lambda )}}{\varphi _{\sigma ,\theta }(w) - \lambda }\right| \le C(\Lambda ,C_w) \end{aligned}$$

or

$$\begin{aligned} \left| \varphi _{\sigma ,\theta }(w) - \lambda \right| \ge \frac{1}{C(\Lambda ,C_w)} \prod _{p_{\lambda } \in {\mathcal {M}}_{\Lambda ,C_w}}{\left| w - p_\lambda \right| }. \end{aligned}$$

Thus, if *w* is separated from ${\mathcal {M}}_\lambda$ and the imaginary axis by $\delta$, we get:

$$\begin{aligned} \left| \varphi _{\sigma ,\theta }(w) - \lambda \right|&\ge \frac{1}{C(\Lambda ,C_w)} \prod _{p_{\lambda } \in {\mathcal {M}}_{\Lambda ,C_w}}{ \underbrace{\left| w - p_\lambda \right| }_{\ge \delta }} \\&\ge \frac{\delta ^{\#{\mathcal {M}}_{\Lambda ,C_w}}}{ C(\Lambda ,C_w)} \frac{\lambda }{\Lambda } =:C(\Lambda ,\delta ,C_w) \lambda . \end{aligned}$$

Here in the last step we used the fact that the number of elements in ${\mathcal {M}}_{\Lambda ,C_w}$ is uniformly bounded, as can be seen from the definition in (A18).

If $\lambda \in [0,\Lambda ]$ and $\left| w\right| > C_w:=\max (\log (2\Lambda /c_1),4\ln (2))$, (where $c_1$ is the constant in (A7) or (A8)) we get:

$$\begin{aligned} \left| \varphi _{\sigma ,\theta }(w)-\lambda \right| \ge \left| \varphi _{\sigma ,\theta }(w)\right| -\lambda \;{\mathop {\ge }\limits ^{{(A7), (A8)}}}\quad \; c_1 e^{\left| {\text {Re}}(w)\right| } - \Lambda \ge \Lambda \ge \lambda . \end{aligned}$$

We therefore may from now on assume that $\lambda$ is sufficiently large as we see fit. In preparation for the rest of the proof, we note that for $\zeta , \mu \in \mathbb {R}$, w.l.o.g., $\left| \zeta \right| \le \left| \mu \right|$:

A19 $$\begin{aligned} \left| \cosh (\mu ) - \cosh (\zeta )\right|= & {} \cosh (\left| \mu \right| )-\cosh (\left| \zeta \right| ) =\int _{\left| \zeta \right| }^{\left| \mu \right| }{\sinh (\tau )\,d\tau }\nonumber \\\ge & {} \sinh (\left| \zeta \right| ) (\left| \mu \right| -\left| \zeta \right| ). \end{aligned}$$

Because it is much simpler, we start with the **case** $\varvec{\sigma }\mathbf {=1}$. We note that in this case ${\mathcal {M}}_\lambda$ consists of the points mapped to $\pm \lambda$. We distinguish three cases, depending on whether ${\text {Re}}(w)$ is small and if ${\text {Im}}(w)$ is close to a pole or not.

***Case 1:*** $(1+\theta )\kappa \cosh ({\text {Re}}(w))< \lambda /2:$ The triangle inequality gives:

$$\begin{aligned} \left| \kappa [\cosh (w)+ i\theta \sinh (w)] - \lambda \right|&\ge \lambda - (1+\theta ) \kappa \cosh ({\text {Re}}(w)) \ge \frac{\lambda }{2}. \end{aligned}$$

***Case 2:*** $2(1+\theta )\kappa \cosh ({\text {Re}}(w)) \ge \lambda$ *and there exists a point* $p \in {\mathcal {M}}_\lambda$ *with* $\left| {\text {Im}}{w} - {\text {Im}}{p}\right| \le \varepsilon _1$ *for* $\varepsilon _1$ *sufficiently small.* We note that this implies that $\left| {\text {Re}}(w)-{\text {Re}}(p)\right|$ is positive. Due to the symmetry in (A11) we may in addition assume that ${\text {sign}}({\text {Re}}(w))={\text {sign}}({\text {Re}}(p))$.

By Lemma A.2 and Lemma A.6(i) we note the following estimates:

A20 $$\begin{aligned} |\sinh ({\text {Re}}(w))| \sim |\cosh ({\text {Re}}(w))| > rsim \lambda , \, \text {and}\, |\sinh ({\text {Re}}(p))| \sim |\cosh ({\text {Re}}(p))| > rsim \lambda . \end{aligned}$$

Writing $h(\eta ):=\cos (\eta )-\theta \sin (\eta )$, we note that $\left| h({\text {Im}}(w))\right|> c> 0$ by (A12) and the fact that adding $\pi \ell$ might only change the sign. If $h({\text {Im}}(p)) \ge 0$, we consider the real part of $\varphi _{\sigma ,\theta }(w)-\lambda$ to get:

$$\begin{aligned}{} & {} \left| \cosh ({\text {Re}}(w))h({\text {Im}}(w))) - \lambda \right| \\{} & {} \quad = \left| \cosh ({\text {Re}}(w))h({\text {Im}}(p)) - \lambda +\cosh ({\text {Re}}(w))\big (h({\text {Im}}(w))-h({\text {Im}}(p))\big )\right| \\{} & {} \quad \ge \left| \cosh ({\text {Re}}(w))h({\text {Im}}(p)) - \lambda \right| - \cosh ({\text {Re}}(w))\left| h({\text {Im}}(w))-h({\text {Im}}(p))\right| \\{} & {} {\mathop { > rsim }\limits ^{{(A19)}}} \min \big (\left| \sinh ({\text {Re}}(w))\right| ,\left| \sinh ({\text {Re}}(p))\right| \big ) \left| {\text {Re}}(w) - {\text {Re}}(p)\right| - (1+\theta )\cosh ({\text {Re}}(w))\varepsilon _1 \\{} & {} {\mathop { > rsim }\limits ^{{(A20)}}}\lambda , \end{aligned}$$

where in the last step we chose $\varepsilon _1$ sufficiently small (but independent of $\lambda$). If $h({\text {Im}}(p))\le 0$, by continuity we can enforce $h({\text {Im}}(w))\le 0$ as long as $\varepsilon _1$ is sufficiently small. The necessary calculation then is even easier because $\varphi _{\sigma ,\theta }(w)$ maps to the left half-plane.

***Case 3:*** $2(1+\theta ) \cosh ({\text {Re}}(w)) \ge \lambda$ *and* $\left| {\text {Im}}(w) - {\text {Im}}(p)\right| \ge \varepsilon _1 > 0$ *for all* $p \in {\mathcal {M}}_\lambda$. We estimate imaginary part of $\varphi _{\sigma ,\theta }$. Since ${\text {Im}}(w)$ has positive distance from the points in (A12), we get $\left| \sin ({\text {Im}}(w))+\theta \cos ({\text {Im}}(w))\right|>c>0$. Which in term gives

$$\begin{aligned} \left| \sinh ({\text {Re}}(w))\right| \left| \sin ({\text {Im}}(w))+\theta \cos ({\text {Im}}(w))\right| \ge c\left| \sinh ({\text {Re}}(w))\right| > rsim \lambda \end{aligned}$$

where the last part only holds for large enough cases of ${\text {Re}}(w)$ not covered by Case 1.

Now we show, how the proof has to be adapted for the **case** $\varvec{\sigma }\mathbf {=1/2}$, again focusing on the asymptotic case of large $\lambda$. By Lemma A.6(i), all the points $p \in {\mathcal {M}}_\lambda$ satisfy $\left| {\text {Re}}(p)\right| \sim \log (\lambda )$. Looking at the imaginary part of the defining equation for *p* we get that

$$\begin{aligned} \left| \cos ({\text {Im}}(p))\right|&=\left| \frac{\sinh ({\text {Re}}(p)/2)}{\theta \cosh ({\text {Re}}(p))} \sin ({\text {Re}}(p))\right| \lesssim \frac{e^{-{\text {Re}}(p)/2} }{\theta } \lesssim \lambda ^{-1/2}. \end{aligned}$$

Thus, for any $\varepsilon _2\in (0,1)$, assuming $\lambda$ is sufficiently large, all the points $p \in {\mathcal {M}}_\lambda$ satisfy $\cos ({\text {Im}}(p))< \varepsilon _2$. We again have to distinguish three cases:

***Case 1:*** $(1+\theta ) \cosh ({\text {Re}}(w))< \lambda /2:$ One can argue just like in the $\sigma =1$ case.

***Case 2:*** $2(1+\theta ) \cosh ({\text {Re}}(w)) \ge \lambda$ *and* *there exists a point* $p \in {\mathcal {M}}_\lambda$ *with* $\left| {\text {Im}}{w} - {\text {Im}}{p}\right| \le \varepsilon _1$ *for* $\varepsilon _1$ *sufficiently small.* We note that this implies that $\left| {\text {Re}}(w)-{\text {Re}}(p)\right|$ is positive. Since $\left| \cos (w)\right| < \varepsilon _2$, we note that $\left| \sin (w)\right| > 1-\varepsilon _2$. By possibly adding $i\ell \pi$, we can write

A21 $$\begin{aligned} \lambda =-\theta \cosh ({\text {Re}}(p))\sin ({\text {Im}}(p+\ell i\pi )) + \cosh ({\text {Re}}(p/2))\sin ({\text {Im}}(p+\ell i\pi )/2). \end{aligned}$$

For large values of $\lambda$, the $\cosh ({\text {Re}}(p))$ term is dominating. Therefore, we have ${\text {sign}}({\text {Re}}(\varphi _{\sigma ,\theta }(p)))=-{\text {sign}}(\sin ({\text {Im}}(p)))$. By continuity we can enforce that ${\text {sign}}(\sin ({\text {Im}}(w)))={\text {sign}}(\sin ({\text {Im}}(p)))$. Since for the case ${\text {Re}}(\varphi _{\sigma ,\theta }(w))\le 0$ the statement is trivial, we only have to consider the case ${\text {sign}}(\sin ({\text {Im}}(w)))=-1$ or $\ell =0$ in (A21). We look at the real part of $\varphi _{\sigma ,\theta }(w)-\lambda$ to get:

$$\begin{aligned}&\big |-\theta \cosh ({\text {Re}}(w))\sin ({\text {Im}}(w)) - \lambda + \cosh ({\text {Re}}(w)/2)\cos ({\text {Im}}(w)/2)\big |\\&\quad \ge \left| -\theta \cosh ({\text {Re}}(w))\sin ({\text {Im}}(p)) + \theta \cosh ({\text {Re}}(p)) \sin ({\text {Im}}(p))\right| \\&\qquad -\left| \cosh ({\text {Re}}(w))\big (\sin ({\text {Im}}(w))-\sin ({\text {Im}}{p})\big )\right| - \mathcal {O}\big (\cosh ({\text {Re}}(w)/2)\big ) \\&\quad > rsim \theta \min \big (\left| \sinh ({\text {Re}}(w))\right| ,\left| \sinh ({\text {Re}}(p))\right| \big ) \left| {\text {Re}}(w) - {\text {Re}}(p)\right| \!-\!C\cosh ({\text {Re}}(w))\varepsilon _1 \\&\quad > rsim \lambda \end{aligned}$$

where we absorbed the term $\cosh ({\text {Re}}(w)/2)$ into $\cosh ({\text {Re}}(w))\varepsilon _1$ by assuming $\lambda$ sufficiently large and in the last step we chose $\varepsilon _1$ sufficiently small (but independent of $\lambda$).

***Case 3:*** $2(1+\theta ) \cosh ({\text {Re}}(w)) \ge \lambda$ *and* $\left| {\text {Im}}{w} - {\text {Im}}{p}\right| \ge \varepsilon _1 > 0$ *for all* $p \in {\mathcal {M}}_\lambda$. Since all the points $p \in {\mathcal {M}}_\lambda$ satisfy $\left| \cos ({\text {Im}}(p))\right| <\varepsilon _2$ and for every zero of $\cos$, there exists a value $p \in {\mathcal {M}}_\lambda$ with ${\text {Im}}(p)$ close to it, this means that $\left| \cos ({\text {Im}}(w))\right| \ge \delta _2$ for a constant $\delta _2$ depending only on $\varepsilon _1$ and $\varepsilon _2$. We estimate

$$\begin{aligned} \left| {\text {Im}}(\varphi _{\sigma ,\theta }(w))\right| \ge \theta \left| \sinh ({\text {Re}}(w))\right| \left| \cos ({\text {Im}}(w))\right| - \left| \cosh (w/2)\right| > rsim \left| \sinh ({\text {Re}}(w))\right| > rsim \lambda \end{aligned}$$

where the last part only holds for large enough cases of ${\text {Re}}(w)$ not covered by the first case. $\square$

### Lemma A.8

Fix $\lambda \ge \lambda _0 > \kappa$. Consider the set

$$\begin{aligned} P_\lambda ^y:=\Big \{ y \in D_{d(\theta )}: \psi _{\sigma ,\theta }(y)=\lambda \Big \}, \end{aligned}$$

where $d(\theta )$ is taken sufficiently small. Then the following holds:

(i) For any $\nu _0 \in [0,d(\theta )]$ and $\delta > 0$ there are at most finitely many points $y_1,\dots ,y_{N_p(\lambda ,\delta )} \in P_\lambda ^y$ satisfying
  $$\begin{aligned} \nu _0-\frac{\delta }{\ln (\lambda /\kappa )}<\left| {\text {Im}}(y_\ell )\right| < \nu _0+\frac{\delta }{\ln (\lambda /\kappa )} \qquad \forall \ell \in 1,\dots , N_p(\lambda ,\delta ). \end{aligned}$$
  The number $N_p(\lambda ,\delta )$ of such points can be bounded by a constant depending only on $\delta$, $\theta$, $\sigma$ and $d(\theta )$, but independently of $\lambda$ and $\nu _0$ .
(ii) For $\lambda \in (\lambda _0,\Lambda )$, one can bound $\displaystyle \left| {\text {Im}}(y)\right| \ge \gamma (\Lambda ) > 0 \; \forall y \in P^y_\lambda ,$ with a constant $\gamma (\Lambda )$ depending on $\Lambda$, $\theta$, $\kappa$, $\lambda _0$. For $\lambda$ sufficiently large, the following asymptotic holds:
  A22 $$\begin{aligned} \left| {\text {Im}}(y)\right| \ge {\left\{ \begin{array}{ll} \frac{\tan ^{-1}(\theta )}{\ln (\lambda /\kappa )} - \mathcal {O}\big (\frac{1}{\ln ^2(\lambda /\kappa )}\big ) &{} \text {if}\, \sigma =1,\\ \frac{\pi }{2\ln (\lambda /\kappa )} - \mathcal {O}\big (\frac{1}{\ln ^2(\lambda /\kappa )}\big ) &{}\text {if}\, \sigma =1/2 \end{array}\right. } \qquad \forall y \in P^y_\lambda , \end{aligned}$$
  where the implied constants depend only on $\theta$, $\kappa$, $\lambda _0$ .
(iii) There exists a parameter $d_{\lambda } \in (d(\theta )/2,d(\theta )]$ and a constant $c>0$ such that
  A23 $$\begin{aligned} \left| \psi _{\sigma ,\theta }(a \pm id_{\lambda }) - \lambda \right| \ge \,c{\lambda /\kappa } \qquad \forall a \in \mathbb {R}. \end{aligned}$$
  *c* depends on $\theta$, $\sigma$ and $\lambda _0$ but is independent of $\lambda$.

### Proof

For now, consider the case $\kappa =1$ and $\lambda _0>1$. Since $\psi _{\sigma ,\theta }(0)=1$, due to continuity, there exists a factor $d(\theta )>0$ and $\varepsilon >0$ such that

$$\begin{aligned} \left| \psi _{\sigma ,\theta }(y)\right|< 1 + \varepsilon<\lambda _0<\lambda \qquad \forall \left| y\right| \le d(\theta ). \end{aligned}$$

By taking $d(\theta )$ at last this small in the definition of $D_{d(\theta )}$ we can exclude the poles satisfying ${\text {Re}}(w)={\text {Re}}(\sinh (y))=0$.

**Proof of **(i): Since $\sinh$ is injective by Lemma A.2(i) we only need to count the points in $H_{\theta }$ which are mapped to $\lambda$ by $\varphi _{\sigma ,\theta }$. Consider a point $w=a+ib$ in the *w* domain and let $\frac{\pi }{2}\sinh (y)=w$ with $y=\xi +i\nu \in D_{d(\theta )}$. Then

$$\begin{aligned} \left| a\right| =\frac{\pi }{2}\left| \sinh (\xi )\right| \left| \cos (\nu )\right| \quad \text { and }\quad \left| b\right|&=\frac{\pi }{2}\left| \cosh (\xi )\right| \left| \sin (\nu )\right| . \end{aligned}$$

Simple computations give $\left| a\right| \tan (\left| \nu \right| ) \le \left| b\right| \le \pi /2 \sin (|\nu |)+\left| a\right| \tan (\left| \nu \right| )$. we only show the second inequality:

$$\begin{aligned} \left| b\right|&=\frac{\pi }{2}\left| \cosh (\xi )\right| \left| \sin (\nu )\right| \le \frac{\pi }{2}\big (1+\left| \sinh (\xi )\right| \big )\left| \sin (\nu )\right| =\frac{\pi }{2}\Big (1+\frac{2|a|}{\pi |\cos (\nu )|}\Big )\left| \sin (\nu )\right| \\&\le \frac{\pi }{2} \sin (|\nu |)+\left| a\right| \tan (\left| \nu \right| ). \end{aligned}$$

By inserting Lemma A.6(i) we get

A24 $$\begin{aligned} \big (\ln (\lambda )-c_1\big ) \tan (\left| \nu \right| ) \le \left| b\right| \le \frac{\pi }{2} \sin (\left| \nu \right| ) +(c_1+\ln (\lambda )) \tan (\left| \nu \right| ). \end{aligned}$$

For the length *L* of this interval, we compute for $\nu _\pm :=\nu _0 \pm \delta \ln (\lambda /\kappa )^{-1}$ (without changing the number of points, we may assume $0\le \nu _- \le \nu _+ \le d(\theta )$):

$$\begin{aligned} L&=\ln (\lambda )\big (\tan (\left| \nu _+\right| )-\tan (\nu _-)\big )+ \frac{\pi }{2} \sin (\nu _+)+c_1\tan (\nu _+) + c_1\tan (\nu _-)\\&\le \ln (\lambda ) \int _{\nu _-}^{{\nu _+}}{\frac{1}{\cos ^2(\tau )}d\tau } + \frac{\pi }{2}+ 2c_1 \le 8 \delta \frac{\ln (\lambda )}{\ln (\lambda )-\ln (\kappa )}+ \frac{\pi }{2}+ 2c_1, \end{aligned}$$

where in the last step we used $\cos (\tau )>1/2$ for $\left| \tau \right| <1/2$ and the definition of $\nu _\pm$. The right-hand side stays uniformly bounded for $\lambda \rightarrow \infty$. Therefore, since the length of this interval is bounded uniformly in $\lambda$, we can apply Lemma A.6(ii) to bound $N_p(\lambda ,\delta )$.

**Proof of **(ii): We only show the asymptotics.

Fix $y\in D_{d(\theta )}$ and write $w:=\frac{\pi }{2}\sinh (y)$. For $0<\left| y\right| <1/2$ it can be easily seen that $\left| y\right| +\frac{2}{3} \left| y\right| ^3> \tan (\left| y\right| ).$ Now, if $\left| {\text {Im}}(y)\right| \ge 2\ln (\lambda )^{-1}$ there is nothing left to show. Otherwise, we can bound

$$\begin{aligned} \left| {\text {Im}}(y)\right|&\ge \left| \tan (y)\right| - \frac{4}{3 \ln (\lambda )^{3}} {\mathop {\ge }\limits ^{{(A24)}}} \frac{\left| {\text {Im}}(w)\right| }{1+c_1 + \ln (\lambda )} - \frac{4}{3 \ln (\lambda )^{3}} \\&\ge \frac{\left| {\text {Im}}(w)\right| }{\ln (\lambda )} - \mathcal {O}\Big (\frac{1}{\ln (\lambda )^2}\Big ). \end{aligned}$$

The result follows from Lemma A.6(i).

**Proof of **(iii): For $d_{\lambda }=d(\theta )$, we can not guarantee that $\psi (\xi +i\,d_\lambda )$ does not hit the value $\lambda$. In this case, we have to modify $d_\lambda$ slightly to get robust estimates. For $d\in \mathbb {R}$, consider the hyperbolas

A25 $$\begin{aligned} \gamma _{d}(\xi ):=\frac{\pi }{2}\sinh (\xi +i\,d) \quad \xi \in \mathbb {R}. \end{aligned}$$

In the light of Lemma A.7 we need to ensure that ${\text {dist}}(\gamma _{d_\lambda },w_p) > rsim 1$ for all $w_p \in {\mathcal {M}}_\lambda$. We will be looking for $d_\lambda$ in a small strip around $d(\theta )$. To simplify notation we define the length

$$\begin{aligned} \omega :=d(\theta ) \frac{\ln (\lambda _0)}{2\ln (\lambda )} \quad \text { such that } \quad d(\theta )/2 \le d(\theta )-\omega \le d(\theta ). \end{aligned}$$

To make things symmetric with respect to the real axis, we consider $\widetilde{{\mathcal {M}}}_{\lambda }:={\mathcal {M}}_{\lambda }- {{\mathcal {M}}}_{\lambda }$. It will therefore be sufficient to focus on the upper right quadrant of the complex plane. All other cases follow by symmetry.

We write $\widetilde{{\mathcal {M}}}^y_\lambda :=\sinh ^{-1}(\frac{2}{\pi }\widetilde{{\mathcal {M}}}_{\lambda })$ for the corresponding points in the *y*-domain. We start by noting that we can easily stay away from the problematic parts of the imaginary axis by making $d(\theta )$ sufficiently small, as if $\left| {\text {Re}}(\sinh (y))\right| <\varepsilon$ we have $\left| {\text {Im}}(\sinh (y))\right| <(1+\varepsilon )\sin ({\text {Im}}(y))$; thus for small real parts we can ensure to fit between $(-b_0,b_0)$ on the imaginary axis. This also means that we only consider points $w_\lambda \in \widetilde{{\mathcal {M}}}_{\lambda }$ with $\left| {\text {Re}}(w_\lambda )\right|>\varepsilon >0$ since our path will have already positive distance to other possible poles.

By (i), the number of points $y_\lambda$ in $\widetilde{{\mathcal {M}}}^y_\lambda$ in the strip $d(\theta )-\omega \le {\text {Im}}(y_\lambda )\le d(\theta )$ can be bounded by a constant *N*, independent of $\lambda$. In order to also avoid points in $\widetilde{{\mathcal {M}}}_\lambda ^y$ which are close but outside the critical strip we also avoid the boundary points $d(\theta )-\omega$ and $d(\theta )$. Since $N+2$ strips of width $\frac{\omega }{2N+4}$ can not cover a strip of width $\omega$, there exists a value $d_{\lambda }$ such that

$$\begin{aligned} d(\theta )-\omega \le d_{\lambda } \le d(\theta ) \quad \text {and} \quad \left| {\text {Im}}(y_{\lambda }) - d_{\lambda }\right| \ge \frac{\omega }{2(N+2)} \quad \forall y_\lambda \in \widetilde{{\mathcal {M}}}^y_\lambda . \end{aligned}$$

For ease of notation, we define $\delta :=\ln (\lambda _0)/(4N+8)$ and note that $\left| {\text {Im}}(y_{\lambda }) - d_\lambda \right| \ge \delta \ln (\lambda )^{-1}$. We show that

$$\begin{aligned} {\text {dist}}(\gamma _{d_\lambda },w_p)&\ge \mu > 0 \qquad \forall w_p \in \widetilde{{\mathcal {M}}}_\lambda \end{aligned}$$

for a constant $\mu > 0$ independent of $\lambda$. We fix $y_p:=\xi _{p}+ i\nu _{p} \in \widetilde{{\mathcal {M}}}_{\lambda }^y$ with $w_{\lambda }=\pi \sinh (y_p)/2$ and a point on $\gamma _{d_\lambda }$ denoted by $y_{\gamma }=\xi _{\gamma }+ d_\lambda i$. We write $s_{p}:={\text {sign}}(\nu _p-d_{\lambda })$ and distinguish two cases: $(\xi _p - \xi _\gamma )s_{p} \le 0$ and $(\xi _p - \xi _\gamma )s_{p}>0$. For symmetry reasons, we only consider the case $\xi _\gamma ,\xi _p,\nu _{p} >0$. **If** $\varvec{(\xi _p - \xi _\gamma )s_{p} \ge 0}$, we get:

$$\begin{aligned}{} & {} {\text {Im}}(s_{p}(\sinh (y_p)-\sinh (y_{\gamma }))) \\{} & {} \quad =s_{p} \big (\cosh (\xi _p)\sin (\nu _p) - \cosh (\xi _{\gamma })\sin (d_{\lambda }) \big )\\{} & {} \quad =\cosh (\xi _p)s_{p}\big (\sin (\nu _p)-\sin (d_{\lambda })\big ) + \underbrace{s_p \Big (\cosh (\xi _p)-\cosh (\xi _{\gamma })\Big )\sin (d_\lambda )}_{\ge 0} \\{} & {} \quad \ge \cosh (\xi _p) s_{p} \int _{d_{\lambda }}^{\nu _p}{\cos (\tau ) d\tau } > rsim \cosh (\xi _p) \frac{\delta }{\ln (\lambda )}. \end{aligned}$$

For the **case** $\varvec{(\xi _p - \xi _\gamma )s_{p} <0}$, we calculate:

A26 $$\begin{aligned}&-s_{p}{\text {Re}}(\sinh (y_{p})-\sinh (y_\gamma ))\nonumber \\&\quad =-s_{p}\sinh (\xi _p)\cos (\nu _p) + s_p\sinh (\xi _\gamma )\cos (d_{\lambda }) \nonumber \\&\quad =-s_{p}\sinh (\xi _p)\Big (\cos (\nu _p)-\cos (d_{\lambda })\big ) + \underbrace{s_{p}\Big (\sinh (\xi _\gamma )-\sinh (\xi _p)\Big )\cos (d_\lambda ))}_{\ge 0} \nonumber \\&\quad \ge \sinh (\xi _p) s_{p} \int _{d_\lambda }^{\nu _p}{\sin (\tau ) d\tau } > rsim \sinh (\xi _p) \sin (d(\theta )/2) \frac{\delta }{\ln (\lambda )}. \end{aligned}$$

We have, since $\sinh (\xi _p) \in \frac{2}{\pi }\widetilde{{\mathcal {M}}}_{\lambda }$ and using Lemma A.6(i) that

$$\begin{aligned} \sinh (\xi _p)&= \frac{{\text {Re}}(\sinh (\xi ))}{\cos (\nu _p)} =\frac{{\text {Re}}(w_{\lambda })}{\cos (\nu _p)} > rsim \frac{\ln (\lambda )-c_1}{\cos (\nu _p)}. \end{aligned}$$

Together with the previous assumption ${\text {Re}}(w_{\lambda })\ge \varepsilon$, this gives $\cosh (\xi _p) \ge \sinh (\xi _p) > rsim \max (\ln (\lambda )-c_1,\varepsilon )$. With this can conclude that for $\ln (\lambda ) > 2c_1\!\!:$

$$\begin{aligned} {\text {dist}}(\gamma _{d_\lambda },w_\lambda ) > rsim \frac{\ln (\lambda )-c_1}{\ln (\lambda )} > rsim 1- \frac{c_1}{2c_1} \ge \frac{1}{2}. \end{aligned}$$

For $\ln (\lambda )<2 c_1$ we get

$$\begin{aligned} {\text {dist}}(\gamma _{d_\lambda },w_\lambda ) > rsim \varepsilon \frac{1}{\ln (\lambda )}> \frac{\varepsilon }{2c_1}>0. \end{aligned}$$

We can now apply Lemma A.7 to get to the final result. The general case $\kappa \ne 1$ follows by dividing the equation $\psi _{\sigma ,\theta }(y)=\lambda$ by $\kappa$. We can therefore just replace $\lambda$ by $\lambda /\kappa$ in all statements. $\square$
